# Supplementary material for: Who are the patients being offered the faecal immunochemical test in routine English general practice, and for what symptoms? A prospective descriptive study
Source: BMJ Open. 2022 Sep 19;12(9):e066051. doi: 10.1136/bmjopen-2022-066051 (PMC9486301; doi:10.1136/bmjopen-2022-066051)
Supplement: Supplementary data [file bmjopen-2022-066051supp001.pdf]

Supplementary data

Who are the patients being offered the faecal immunochemical test in routine English general practice, and for what symptoms? A prospective descriptive study

Online supplemental file 1. Patient questionnaire

Section 1: About Your Symptoms
We are interested in symptoms which you think are related to your recent poo test request. Did you have any of the following symptoms or health problems?

1. Change in bowel habit
☐ Yes ☐ No If YES please indicate the severity:
☐ Mild ☐ Moderate ☐ Quite a lot ☐ Severe ☐ Very severe ☐ Unbearable

2. Change in bowel habit – mainly diarrhoea or loose poo
☐ Yes ☐ No If YES please indicate the severity:
☐ Mild ☐ Moderate ☐ Quite a lot ☐ Severe ☐ Very severe ☐ Unbearable

3. Change in bowel habit – mainly constipation
☐ Yes ☐ No If YES please indicate the severity:
☐ Mild ☐ Moderate ☐ Quite a lot ☐ Severe ☐ Very severe ☐ Unbearable

4. Abdominal pain or discomfort that wasn't normal for you
☐ Yes ☐ No If YES please indicate the severity:
☐ Mild ☐ Moderate ☐ Quite a lot ☐ Severe ☐ Very severe ☐ Unbearable

5. Bleeding from your back passage
☐ Yes ☐ No If YES please indicate the severity:
☐ Mild ☐ Moderate ☐ Quite a lot ☐ Severe ☐ Very severe ☐ Unbearable

6. Indigestion or heartburn
☐ Yes ☐ No If YES please indicate the severity:
☐ Mild ☐ Moderate ☐ Quite a lot ☐ Severe ☐ Very severe ☐ Unbearable

7. Bloating
☐ Yes ☐ No If YES please indicate the severity:
☐ Mild ☐ Moderate ☐ Quite a lot ☐ Severe ☐ Very severe ☐ Unbearable

8. Fatigue or tiredness that is unusual for you
☐ Yes ☐ No If YES please indicate the severity:
☐ Mild ☐ Moderate ☐ Quite a lot ☐ Severe ☐ Very severe ☐ Unbearable

9. Unexplained weight loss
☐ Yes ☐ No If YES please indicate the severity:
☐ Mild ☐ Moderate ☐ Quite a lot ☐ Severe ☐ Very severe ☐ Unbearable

10. Loss of appetite
☐ Yes ☐ No If YES please indicate the severity:
☐ Mild ☐ Moderate ☐ Quite a lot ☐ Severe ☐ Very severe ☐ Unbearable

11. Mucous or slime in poo
☐ Yes ☐ No If YES please indicate the severity:
☐ Mild ☐ Moderate ☐ Quite a lot ☐ Severe ☐ Very severe ☐ Unbearable

Section 2: About You
12. How would you describe your ethnicity? Please tick one box only
White

☐ British ☐ Irish
☐ Any other white background. Please write...

Mixed
☐ White and Black Caribbean ☐ White and Black African ☐ White and Asian
☐ Any other Mixed background. Please write...

Chinese or other ethnic group
☐ Chinese ☐ Any other ethnic group. Please write...

Asian or Asian British
☐ Indian ☐ Pakistani ☐ Bangladeshi ☐ Any other Asian background. Please write................................

Black or Black British
☐ Caribbean ☐ African ☐ Any other Black background. Please write...

13. What is your highest level of qualification? Please tick one box only
☐ Degree (or equivalent) ☐ Other higher education ☐ A-Level (or equivalent)
☐ GCSE/O-Level (or equivalent) ☐ None ☐ Other, please specify.................................

14. Do you use any of the following medications?
☐ NSAID/Anti-inflammatory painkillers ☐ Aspirin
☐ Anticoagulants/blood thinners (e.g. warfarin, coumarines) ☐ Antidepressants

15. Are you suffering from, or have you suffered from, any of the following? Please tick any that are applicable to you
☐ Inflammatory bowel disease (e.g. Crohn's or colitis) ☐ Diabetes o Irritable bowel syndrome
☐ Arthritis ☐ Peptic ulcer ☐ Anxiety or depression o Bowel (colon or rectal) cancer ☐ Bowel polyps
☐ Other cancer. Please write which type...

16. Have you had a colonoscopy, sigmoidoscopy or bowel scope?
In the past 5 years? ☐ Yes ☐ No
Ever? ☐ Yes ☐ No

17. Have you had a bowel screening test or poo test?
In the past 5 years? ☐ Yes ☐ No
Ever? ☐ Yes ☐ No

18. Smoking - please tick one box only. Are you?
☐ A current smoker? ☐ An ex-smoker? ☐ A non-smoker (never smoked)?

19. Alcohol. How often have you had an alcoholic drink of any kind in the last 12 months? Please tick one
☐ Almost every day ☐ About once a week ☐ About twice a week ☐ About once a fortnight
☐ Only a few times a year ☐ I never drink alcohol

20. What is your height (fill in one of the options)?
In Feet and Inches or CM...

21. What is your current weight (fill in one of the options)?
Stones and pounds..Kg...

22. Have any of your close relatives, including parent(s), children, brother(s) or sister(s), had colon or rectal cancer (also known as large bowel or colorectal cancer) before the age of 55?
☐ Yes ☐ No

23. Do you have more than one relative who has had colon or rectal cancer on either your mother's or father's side of the family? Please think about all of the following relatives: parent(s), children, brother(s), sister(s), grandparent(s), aunt(s), uncle(s), niece(s), nephew(s) and grandchildren.
☐ Yes ☐ No
