# Supplementary material for: Who are the patients being offered the faecal immunochemical test in routine English general practice, and for what symptoms? A prospective descriptive study
Source: BMJ Open. 2022 Sep 19;12(9):e066051. doi: 10.1136/bmjopen-2022-066051 (PMC9486301; doi:10.1136/bmjopen-2022-066051)
Supplement: Supplementary data [file bmjopen-2022-066051supp002.pdf]

Supplementary data

Who are the patients being offered the faecal immunochemical test in routine English general practice, and for what symptoms? A prospective descriptive study

Supplemental file 2. FIT requests and GP-recorded symptoms in the East of England between November 2018 and May 2020

Anonymised data on FIT requests, FIT results and GP-recorded symptom/s with FIT request covering the period from FIT implementation in November 2018 to May 2020.

From November 2018 to May 2020, 15,620 valid FITs were performed in this region. The number of FITs ordered increased from November 2018 to May 2020, reaching a peak in January 2020 (1512 valid tests), reducing during the early pandemic (compared with January, March, 26.3% and April, 50.1% decreases respectively), and increasing again in May 2020 (1465 valid tests) (Figure 1). Of the 15,620 valid FITs performed during this period, 20.7% (n=3237) were positive (range 15.1% (November 2018, Norfolk only) to 29.1% (both areas, April 2020)).

Figure 1. All FIT requests and FIT positives in Eastern England post DG30 implementation

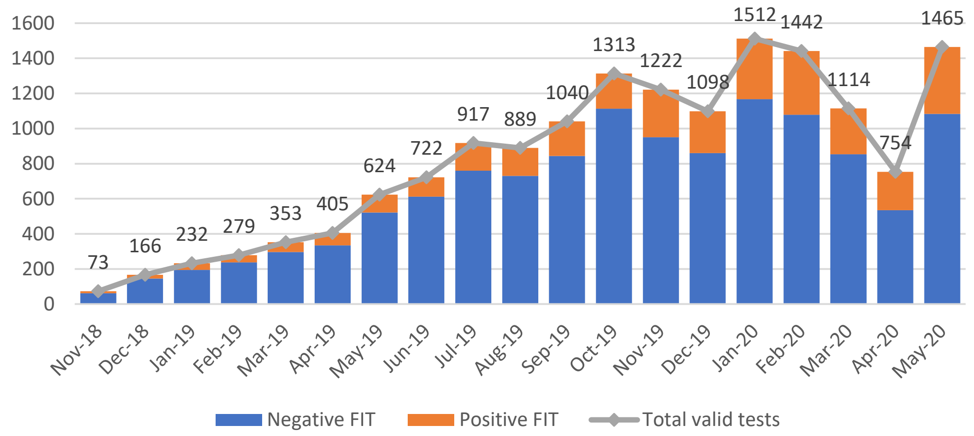

The proportion of GP-recorded symptoms was lower when compared to patient-reported symptoms, and similar to GP-recorded symptoms in the study. This was the case for all reported symptoms (Figure 2).

Figure 2. Historical and FIT-East data for symptom reporting

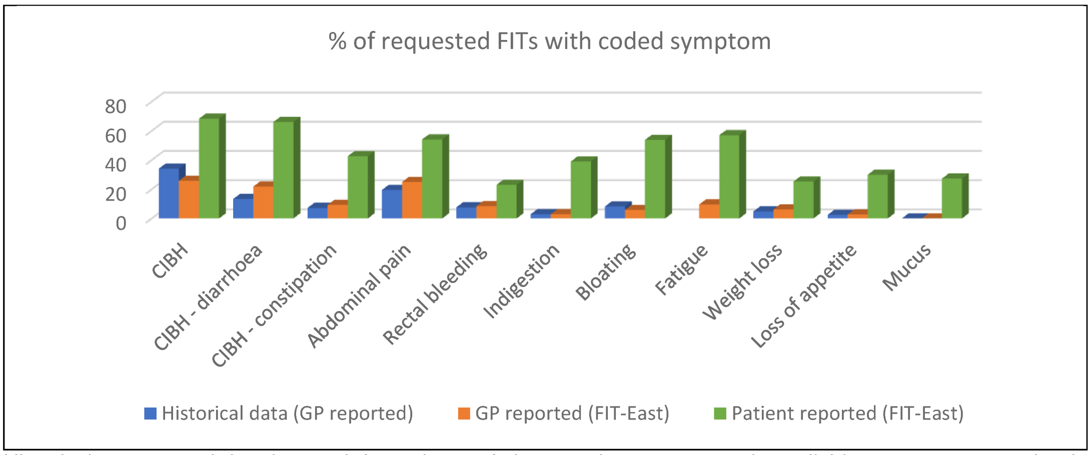

Historical, aggregated data has no information on fatigue, and mucus was only available as an answer option in the patient questionnaire.
