# Supplementary material for: Who are the patients being offered the faecal immunochemical test in routine English general practice, and for what symptoms? A prospective descriptive study
Source: BMJ Open. 2022 Sep 19;12(9):e066051. doi: 10.1136/bmjopen-2022-066051 (PMC9486301; doi:10.1136/bmjopen-2022-066051)
Supplement: Supplementary data [file bmjopen-2022-066051supp004.pdf]

Supplementary data

Who are the patients being offered the faecal immunochemical test in routine English general practice, and for what symptoms? A prospective descriptive study

Supplemental table 1. Patient characteristics according to availability of linked data

Characteristics	Linked data available (full dataset) (n=310)	Linked data not available (n=197)	Tests
Age			
Median age (IQR)	70 (61-77)	66 (53.5-75.0)	**U=36222.5, *p*<0.001**
Gender	n (%)	n (%)	
Female	160 (53)	100 (52)	
Male	142 (47)	93 (48)	χ(1)=0.064, *p*=0.80
Deprivation (IMD Quintiles)	n (%)	n (%)	
1 (least deprived)	69 (22)	30 (18)	**U=22457, *p*=0.04**
2	74 (24)	26 (16)	
3	88 (28)	55 (34)	
4	49 (16)	34 (21)	
5 (most deprived)	30 (10)	18 (11)	
