# Supplementary material for: Who are the patients being offered the faecal immunochemical test in routine English general practice, and for what symptoms? A prospective descriptive study
Source: BMJ Open. 2022 Sep 19;12(9):e066051. doi: 10.1136/bmjopen-2022-066051 (PMC9486301; doi:10.1136/bmjopen-2022-066051)

Supplementary data

Who are the patients being offered the faecal immunochemical test in routine English general practice, and for what symptoms? A prospective descriptive study

Supplemental figure 1. Patient-reported symptom patterns

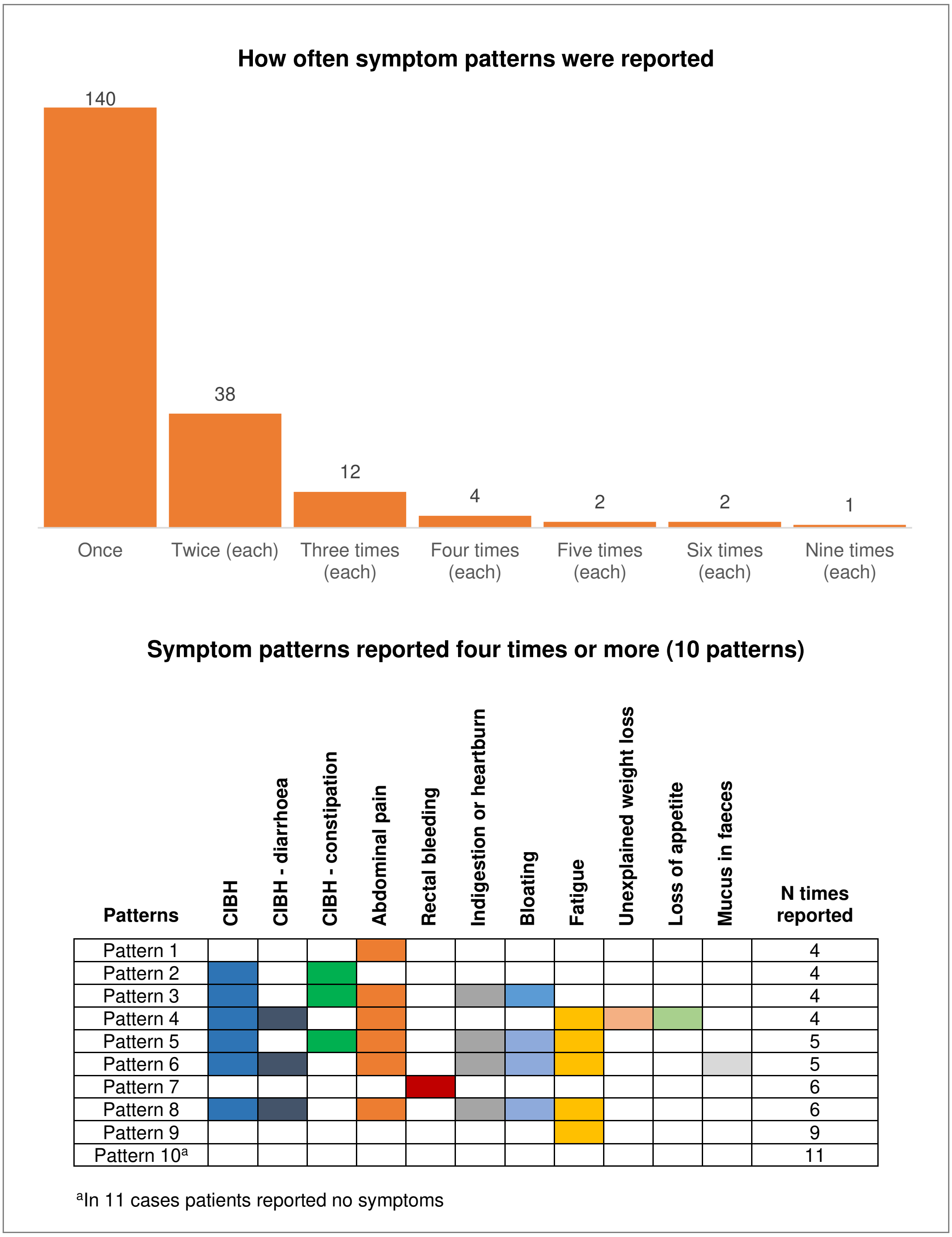

Supplement: Supplementary data [file bmjopen-2022-066051supp003.pdf]
