# Supplementary material for: Who are the patients being offered the faecal immunochemical test in routine English general practice, and for what symptoms? A prospective descriptive study
Source: BMJ Open. 2022 Sep 19;12(9):e066051. doi: 10.1136/bmjopen-2022-066051 (PMC9486301; doi:10.1136/bmjopen-2022-066051)
Supplement: Author's manuscript [file bmjopen-2022-066051.draft_revisions.pdf]

BMJ Open is committed to open peer review. As part of this commitment we make the peer review history of every article we publish publicly available.

When an article is published we post the peer reviewers' comments and the authors' responses online. We also post the versions of the paper that were used during peer review. These are the versions that the peer review comments apply to.

The versions of the paper that follow are the versions that were submitted during the peer review process. They are not the versions of record or the final published versions. They should not be cited or distributed as the published version of this manuscript.

BMJ Open is an open access journal and the full, final, typeset and author-corrected version of record of the manuscript is available on our site with no access controls, subscription charges or pay-per-view fees (http://bmjopen.bmj.com).

If you have any questions on BMJ Open's open peer review process please email info.bmjopen@bmj.com

BMJ Open

Who are the patients being offered the faecal immunochemical test in routine English general practice? A prospective descriptive study

Journal:	*BMJ Open*
Manuscript ID	bmjopen-2022-066051
Article Type:	Original research
Date Submitted by the Author:	24-Jun-2022
Complete List of Authors:	Calanzani, Natalia ; University of Cambridge, Department of Public Health and Primary Care<br>Pannebakker, Merel M; University of Cambridge, Department of Public Health and Primary Care<br>Tagg, Max J; University of Cambridge<br>Walford, Hugo; University College London<br>Holloway, Peter; East of England Cancer Alliances<br>de Wit, Niek; University Medical Center Utrecht, Julius Center for Primary Care<br>Hamilton, Willie; University of Exeter, Primary Care Diagnostics<br>Walter, Fiona M; Queen Mary University of London; University of Cambridge, Department of Public Health and Primary Care
Keywords:	Adult gastroenterology < GASTROENTEROLOGY, Adult oncology < ONCOLOGY, Gastrointestinal tumours < ONCOLOGY, PRIMARY CARE

SCHOLARONE™
Manuscripts

I, the Submitting Author has the right to grant and does grant on behalf of all authors of the Work (as defined in the below author licence), an exclusive licence and/or a non-exclusive licence for contributions from authors who are: i) UK Crown employees; ii) where BMJ has agreed a CC-BY licence shall apply, and/or iii) in accordance with the terms applicable for US Federal Government officers or employees acting as part of their official duties; on a worldwide, perpetual, irrevocable, royalty-free basis to BMJ Publishing Group Ltd ("BMJ") its licensees and where the relevant Journal is co-owned by BMJ to the co-owners of the Journal, to publish the Work in this journal and any other BMJ products and to exploit all rights, as set out in our licence.

The Submitting Author accepts and understands that any supply made under these terms is made by BMJ to the Submitting Author unless you are acting as an employee on behalf of your employer or a postgraduate student of an affiliated institution which is paying any applicable article publishing charge ("APC") for Open Access articles. Where the Submitting Author wishes to make the Work available on an Open Access basis (and intends to pay the relevant APC), the terms of reuse of such Open Access shall be governed by a Creative Commons licence – details of these licences and which Creative Commons licence will apply to this Work are set out in our licence referred to above.

Other than as permitted in any relevant BMJ Author's Self Archiving Policies, I confirm this Work has not been accepted for publication elsewhere, is not being considered for publication elsewhere and does not duplicate material already published. I confirm all authors consent to publication of this Work and authorise the granting of this licence.

For peer review only

Who are the patients being offered the faecal immunochemical test in routine English general practice? A prospective descriptive study

Natalia Calanzani[1], Merel M Pannebakker[1], Max J Tagg[2], Hugo Walford[3], Peter Holloway[4], Niek de Wit[5], Willie Hamilton[6], Fiona M Walter[7,1,8]

[1]The Primary Care Unit, Department of Public Health and Primary Care, University of Cambridge, Cambridge, UK, ORCID ID: 0000-0002-5068-2543 (NC)
[2]School of Clinical Medicine, Addenbrooke's Hospital, University of Cambridge, UK, ORCID ID: 0000-0003-0341-3284
[3]School of Clinical Medicine, University College London, UK, ORCID ID: 0000-0001-9655-9674
[4]East of England Cancer Alliances, UK
[5]Julius Center for Health Sciences and Primary Care, University Medical Center, Utrecht University, Netherlands
[6]University of Exeter Medical School, Exeter, UK, ORCID ID: 0000-0003-1611-1373
[7]Wolfson Institute of Population Health, Queen Mary University of London, UK, ORCID ID: 0000-0002-7191-6476
[8]Centre for Cancer Research and Department of General Practice, University of Melbourne, Melbourne, Australia

Corresponding author:
Dr Natalia Calanzani, nm719@medschl.cam.ac.uk
The Primary Care Unit, Department of Public Health and Primary Care
Strangeways Research Laboratory, 2 Worts Causeway
University of Cambridge
Cambridge, CB1 8RN

Word count: 2790

For peer review only - http://bmjopen.bmj.com/site/about/guidelines.xhtml

Abstract

Objectives: The faecal immunochemical test (FIT) was introduced in 2018 to triage patients with lower-risk symptoms of colorectal cancer (CRC) in English primary care. While there is growing evidence on its utility to triage patients in this setting, evidence is still limited on how official FIT guidance is being used, for which patients, and for what symptoms. We aimed to investigate how FIT was being used routinely in primary care practice.

Design: A prospective, descriptive study of patients offered a FIT in primary care between January and June 2020.

Setting: East of England general practices.

Participants: Consenting patients (aged ≥40 years) who were seen by their general practitioners (GPs) with symptoms of possible CRC for whom a FIT was requested. We excluded patients receiving a FIT for screening purposes, or patients deemed by GPs as lacking capacity for informed consent. Data were obtained via patient questionnaire, medical and laboratory records.

Primary and secondary outcome measures: FIT results (10 µg Hb/g faeces defined a positive result); patient sociodemographic and clinical characteristics; patient-reported and GP-recorded symptoms, symptom severity and symptom agreement between patient and GP (% and kappa statistics).

Results: Complete data were available for 310 patients, median age 70 (IQR 61-77), 53% female, and 23% FIT positive. Patients most commonly reported change in bowel habit (69%) and fatigue (57%) while GPs most commonly recorded abdominal pain (25%) and change in bowel habit (24%). Symptom agreement ranged from 44% (fatigue) to 80% (unexplained weight loss). Kappa agreement was universally low across symptoms.

Conclusion: Almost a quarter of this primary care cohort of symptomatic patients with FIT testing were found to be positive. However, there was low agreement between patient-reported and GP-recorded symptoms. This may impact cancer risk assessment and optimal patient management in primary care.

For peer review only - http://bmjopen.bmj.com/site/about/guidelines.xhtml

Keywords

Primary care, gastrointestinal tumours, adult oncology, adult gastroenterology

Strengths and limitations of this study

- Recent data on how FIT is used in primary care, and which symptoms trigger a FIT request
- To our knowledge, this is the first study in England to report on the agreement between patient-reported and GP-recorded symptoms
- Limitations include poor access to primary care records, and consequently limited evidence on cancer diagnoses
- There was over-representation of White British populations; therefore results may not be generalisable to different groups

For peer review only

Introduction

Colorectal cancer (CRC) is the second most common cause of cancer deaths worldwide and in the UK.[1] Despite screening programmes, most CRCs are diagnosed after symptomatic presentation,[2] with patients usually presenting first in primary care.[3] Those presenting with alarm symptoms equating to a CRC risk of ≥3% can be referred via an urgent suspected cancer pathway, following the National Institute for Health and Care Excellence (NICE) NG12 guidelines.[4] Recognising that patients with lower-risk ("low risk, but not no-risk'')[5] symptoms may also have cancer, more recent NICE guidance (DG30) recommends offering a faecal immunochemical test (FIT) to patients "without rectal bleeding who have unexplained symptoms but do not meet criteria for a suspected cancer pathway".[6] Those with results ≥10 μg Hb/g faeces are recommended to be referred for further investigations.

The evidence review that informed DG30 development did not identify any studies using FIT in primary care before referral (i.e. as a triage tool),[7] raising the risk of spectrum bias (variation in performance due to testing in different populations).[8] However, since implementation of this guidance, several studies in primary care (including patients with higher-risk and/or lower-risk symptoms)[9-17] have demonstrated that FIT is an effective tool for CRC in primary care, helping to reassure patients and professionals, and optimising referrals for definitive investigations. Some have also suggested the use of different FIT thresholds for patients with different levels of risk.[9, 10, 17] Due to the COVID-19 pandemic, use of FIT has recently been intensified, including to prioritise urgent referrals.[18]

A comprehensive analysis of diagnostic performance of FIT in Southwest England showed that FIT performs exceptionally well to triage patients presenting in primary care with lower-risk CRC symptoms (as specified by DG30 guidance).[9] However, evidence is still limited on how guidance is being used in practice, and for which patients. There is also little evidence about the presenting symptom/s that prompt patients to consult and general practitioners (GPs) to request a FIT. The aim of this study was to investigate use of FIT in English primary care for patients with possible symptoms of CRC. We report on the characteristics of patients for whom a FIT was requested, and agreement between patient-reported and GP-recorded symptoms.

Methods

Design

We conducted a pragmatic, prospective, descriptive study in the East of England, where a quantitative FIT service was launched in primary care from late 2018.

For peer review only - http://bmjopen.bmj.com/site/about/guidelines.xhtml

Setting and population

The study was set in all Eastern Cancer Alliance General Practices in two of six Sustainability and Transformation Plans (STPs): Suffolk & North East Essex and Norfolk & Waveney (estimated adult population of 1,160,215).[19]

We included adult patients aged ≥40 years who were seen by their GPs with symptoms of possible CRC between January and June 2020, but who were not urgently referred for investigation (NICE NG12),[4, 6] and for whom a FIT was requested. We excluded patients receiving a FIT for screening purposes, or patients deemed by GPs as lacking capacity for informed consent.

Two laboratories responsible for all the FITs added study recruitment letters, consent forms and a prepaid envelope to FIT kits distributed to GP practices. These were given to patients when a FIT was requested.

Data sources

Study data

Data collection started on 24 January 2020 (kits sent from laboratories to GP practices) and ceased on 31 January 2021 (data obtained from primary care records). Patient consent forms were only accepted until 30 June 2020 due to clinical pathway changes resulting from the COVID-19 pandemic (i.e., FIT being increasingly performed in primary care for high-risk patients who before the pandemic would meet criteria for urgent referral).

Patients were asked about their age, gender and postcode (to calculate deprivation quintiles) on consent forms and invited to complete a questionnaire (Supplementary File 1) by post or online (Qualtrics, Provo, UT). A reminder was sent after two weeks. The questionnaire was developed from the SYMPTOM questionnaire[20-22] and the Family History Questionnaire (FHQ-12).[23, 24] It included items on symptoms (change in bowel habit, change in bowel habit with mainly diarrhoea, change in bowel habit with mainly constipation, abdominal pain, rectal bleeding, indigestion/heartburn, bloating, fatigue, unexplained weight loss, loss of appetite and mucus in faeces), symptom severity (Likert-type items, from mild (1) to unbearable (6)), socio-demographic characteristics and lifestyle behaviours, medication use, medical history and family history of CRC.

The laboratories also collected FIT and blood test results on consented patients. The FIT assays used were OC-Sensor (Norfolk) and HM-JACKarc (Suffolk): a ‘positive’ test was

For peer review only - http://bmjopen.bmj.com/site/about/guidelines.xhtml

defined as fHb ≥10 µg Hb/g faeces, as per NICE DG30[6]. Results using the OC-Sensor were presented in ng/ml, which were then converted into µg Hb/g by dividing the results by 5. Additionally, laboratories provided GP-recorded symptom data, collected via the FIT request form which had been adapted with the Eastern Cancer Alliance, to match symptoms in the patient questionnaire (except for additional questions about mucus in faeces and symptom severity which were only available for patients).

Finally, primary care records were accessed by practice staff or remotely by the research team (if approved by practices) ≥6 months after FIT was requested to collect clinical outcome data (medical history and cancer diagnoses). These data were only available for a third of patients as practices had to be contacted about each consenting patient – resulting in unfeasible additional workload for many primary care practices during the COVID-19 pandemic. It is unlikely that these data were missing at random and clinical outcome data are only reported descriptively to avoid misleading results – no measures of diagnostic performance were therefore calculated.

Data on patient age, gender and postcode from consent forms were cross-checked when provided by more than one data source (patient responses were prioritised). A database was created in Microsoft Access 2016 (secure server, password protected) for data collection and recording of key data, study timelines and information on data completeness.

Historical data for comparison purposes

Anonymised historical, aggregated data on FIT requests, FIT results and GP-recorded symptom/s with FIT request were provided by the North East Essex and Suffolk Pathology Services and the NHS Ipswich and East Suffolk Clinical Commissioning Group. These data covered the period from FIT implementation (test launch in primary care) in November 2018 to May 2020, for the whole of East of England. This was important for comparison purposes, and for assessing relevance of study population/symptomatology.

Data analysis

Data from consented patients were anonymised and exported into SPSS v.27 format. Analyses were carried out for patients with data from questionnaires, for whom there were also FIT results and GP-reported symptom data. This ensured a full, complete dataset.

Body mass index (BMI) was calculated using patient self-reported values (kg/m^2). Practice postcodes were used to calculate deprivation when patient postcodes were missing. All other

For peer review only

For peer review only - http://bmjopen.bmj.com/site/about/guidelines.xhtml

missing data were coded as such, using the same code/label to ensure consistency across different variables and datasets.

Descriptive statistics (frequencies, percentages and measures of central tendency) were used for patient self-reported data, GP-recorded symptoms, symptom severity and test results. Agreement between symptoms reported by patients and recorded by GPs was calculated as the number of agreed upon symptoms (both patient-reported and GP-recorded) divided by the sum of cases with agreements and the cases with disagreements. Kappa statistics were calculated and interpreted as poor (<0.00) slight (0.00-0.20), fair (0.21-0.4), moderate (0.41-0.60), substantial (0.61-0.80) or almost perfect (0.81-1.00) agreement.[25]

Patient and public involvement statement

There was no patient and public involvement in this analysis. However, the study also includes a qualitative component to assess views on FIT experience and acceptability (reported separately) with PPI involvement (specifically, reviewing and analysing a subsection of interview transcripts).

Results

Population and setting

From January to June 2020, 560 patients from in Suffolk & North East Essex and Norfolk & Waveney (average 108 per month) consented to take part in the study.

Historical data (Supplementary file 2) for the first half of 2020 (January to May 2020) show that there were 6287 FIT requests in the whole South East of England (average 1257 per month with variation during the COVID-19 pandemic).

Patient flow

507 consenting patients (91%) were eligible for inclusion ("initial sample"). Linked data from all sources ("full dataset" with patient data (questionnaire), lab records and GP recording of symptoms for each patient) were available for 310 (61%) linked patients (Figure 1).

[Insert Figure 1 here]

Patient characteristics

In the full dataset (n=310), almost a quarter of FIT results were positive (23%, n=72). Historical data show a similar proportion of positive FITs for the East of England from November 2018

For peer review only - http://bmjopen.bmj.com/site/about/guidelines.xhtml

(test launch) to May 2020 (15,620 valid FITs were performed overall, 21% (n=3237) positive) (Supplementary File 2).

Patient median age was 70 (IQR 61-77) and over half were female (53%) (Table 1). About a quarter of patients (24%) lived in areas of higher deprivation. Compared to the initial sample (n=507), the full dataset was representative in most characteristics, but the most deprived and older patients were underrepresented (Supplementary Table 1).

Table 1. Sociodemographic characteristics of sample

	**Full dataset (n=310)**
**Available for all in the initial sample**	
Age	
Median age (IQR)	70 (61-77)
Gender	n (%)
Female	160 (53)
Male	142 (47)
Deprivation (IMD Quintiles)	
1 (least deprived)	70 (23)
2	73 (24)
3	88 (28)
4	49 (16)
5 (most deprived)	30 (10)
**Questionnaire data**	
Ethnicity	n (%)
White British	289 (94)
White non-British[a]	13 (4)
Other[b]	7 (2)
Education	
Higher education	110 (37)
A-Level or equivalent	33 (11)
GCSE/O-level or equivalent	79 (26)
Other[c]	18 (6)
None	60 (20)
Family history	
Close relatives with CRC before the age of 55	17 (66)
More than one relative with CRC	40 (13)

Percentages may not add up to 100% due to rounding. Missing data: gender (full dataset n=8, 2.6%; initial dataset n=12, 2.4%); ethnicity (n=1, 0.3%), education (n=11, 3.2%). Percentages in table calculated considering complete cases. [a]White Irish (n=7), any other White background (n=6). [b]Not further described to protect patient anonymity as n<5 for each). [c]Any other qualification, e.g. “Diploma”, “High School”, “Police Promotion Exams, “Teacher”, etc.

Table 2 shows that half the sample were non-smokers, 44% were frequent drinkers, over 50% were either overweight or obese, and almost three-quarters had a bowel screening test in the past five years. The most common non-gastrointestinal comorbidities were arthritis and anxiety or depression, and most blood test results were normal.

Table 2. Patient lifestyle, medication use and co-morbidities

	**Full dataset (n=310)**

For peer review only - http://bmjopen.bmj.com/site/about/guidelines.xhtml

For peer review only

	n (%)
**Questionnaire data**	
Smoking status	
Current smoker	23 (7)
Ex-smoker	125 (40)
Non-smoker (never smoked)	162 (52)
Alcohol in last 12 months	
Frequent drinker	138 (45)
Infrequent drinker	48 (16)
Teetotal/rarely drinks	124 (40)
BMI	
Underweight (below 18.5)	12 (4)
Normal (18.5-24.9)	135 (44)
Overweight (25-29.9)	101 (33)
Obese (30 and above)	56 (18)
Imaging and screening	
Bowel screening test (past 5 years)	226 (73)
Medication use [26]	
NSAIDs	59 (19)
Aspirin	47 (15)
Anticoagulants/blood thinners	51 (17)
Antidepressants	38 (12)
None of the above	145 (47)
Co-morbidities (non-GI only)	
Diabetes	43 (14)
Arthritis	95 (31)
Anxiety or depression	77 (25)
**Laboratory data**[b]	
Hb[c] (n=282)	
Low	51 (18)
Within normal range	231 (82)
Platelet count[d] (n=282)	
Low	11 (4)
Within normal range	254 (90)
High (>400)	17 (6)
ESR[e] (n=53)	
Low	3 (6)
Within normal range	32 (60)
High	18 (34)
CRP[f] (n=190)	
Within normal range	142 (75)
High	48 (25)

Percentages may not add up to 100% due to rounding. BMI: Body mass index. NSAIDS: Nonsteroidal anti-inflammatory drugs. Hb: Haemoglobin. ESR: Erythrocyte sedimentation rate. CRP: C-Reactive protein. Missing data: BMI (n=6). Percentages in table calculated considering complete cases. [a]Frequent drinker: almost every day or about twice a week. Infrequent drinker: about once a week or about once a fortnight. Teetotal/rarely drinks: only a few times a year or never drinks alcohol.[b]4 cases with available test results were excluded as they were not carried out ≤6 months of a FIT (n=3) or dates were not available (n=1) – these tests are more likely to have been carried out for different reasons. Median interval between FIT and other tests was 2 days (IQR 6.5-). [c]Hb ranges: low <130 males and <115 females, within normal range 130-180 males and 115-165 females. [d]Platelet ranges: low <140, within normal range 140-400, high >400. [e]ESR ranges: low <1 males and <3 females, within normal range 1-10 males and 3-15 females, high >10 males and >5 females. [f]CRP within normal range 0-5, high >5.

Symptoms reported by patients and recorded by their GPs

Commonly reported symptoms

The commonest patient-reported symptoms were change in bowel habit (69%) and fatigue (57%) while the least common was rectal bleeding (23%). In comparison, the commonest GP-recorded symptoms were abdominal pain (25%) and change in bowel habit (24%) while the least common was loss of appetite (3%) (Table 3). Two hundred different patient-reported

For peer review only - http://bmjopen.bmj.com/site/about/guidelines.xhtml

symptom patterns were identified; most of them (n=140) were reported only once. The maximum number of times a symptom pattern was reported was nine times (fatigue only), or 11 times if having no symptoms is considered a pattern (Supplementary Figure 1).

Historical data for the East of England also show that abdominal pain (20%) and change in bowel habit (35%) were the commonest GP-recorded symptoms while loss of appetite was also the least common (3%) (Supplementary File 2).

Patient- and GP- agreement

Agreement between patient-reported and GP-recorded symptoms ranged from 44% for fatigue to 80% for unexplained weight loss. Kappa agreement was universally low across symptoms (lowest for bloating, fatigue and loss of appetite). GPs recorded symptoms less often, and recorded fewer symptoms per patient (median 1 symptom IQR 1-1), compared to patients (median 5, IQR 3-6 – excluding mucus in faeces) (Table 3).

Similarly, historical data for the whole of East of England show that the proportion of GP-recorded symptoms was lower when compared to patient-reported symptoms, for all symptoms (Supplementary File 2).

Table 3. Patient-reported and GP- recorded symptoms and agreement

	**Full dataset (n=310)**					
	**Patient-reported[a]**	**GP-recorded[b]**	**Reported by patient but not recorded by GP**	**Recorded by GP but not reported by patient**	**Agreement**	**Kappa (95% CIs)[c]**
	n (%)	n (%)	n (%)	n (%)	%	
Change in bowel habit (CIBH)	213 (69)	75 (24)	146 (47)	8 (3)[d]	50	0.167 (0.100 - 0.234)
CIBH: diarrhoea	139 (45)	72 (23)	77 (25)	10 (3)[e]	72	0.406 (0.312 - 0.500)
CIBH: constipation	93 (30)	32 (10)	69 (22)	8 (3)[f]	75	0.272 (0.164 - 0.380)
Abdominal pain	170 (55)	77 (25)	114 (37)	21 (7)	57	0.169 (0.080 - 0.257)
Rectal bleeding	72 (23)	21 (7)	60 (19)	9 (3)	78	0.171 (0.057 - 0.285)
Indigestion / heartburn	128 (41)	11 (4)	119 (38)	2 (1)	61	0.069 (0.014 - 0.124)
Bloating	170 (55)	16 (5)	158 (50)	4 (1)	48	**0.038** (0.005 - 0.081)
Fatigue	178 (57)	35 (11)	158 (50)	15 (5)	44	**-0.001** (-0.064 - 0.062)
Unexplained weight loss	76 (25)	23 (7)	57 (18)	4 (1)	80	0.305 (0.187 - 0.423)
Loss of appetite	89 (29)	8 (3)	85 (27)	4 (1)	71	**0.037** (-0.0305 - 0.100)
Mucus in faeces	85 (28)	N/A	N/A	N/A	N/A	N/A

For peer review only - http://bmjopen.bmj.com/site/about/guidelines.xhtml

Percentages may not add up to 100% due to rounding. [a]Median interval between FIT request being processed by the lab and the patient returning the questionnaire where symptoms were reported was 42 days (IQR 30-56). [b]GPs recorded symptoms when requesting a FIT. [c]Kappa interpretation: poor (<0.00) slight (0.00-0.20), fair (0.21-0.4), moderate (0.41-0.60), substantial (0.61-0.80) or almost perfect (0.81-1.00) agreement. Significant p-values indicate that agreement is significantly different from what would be achieved by chance. [d]In all these cases patient had not reported change in bowel habit, diarrhoea nor constipation. [e]In five of these cases patient had reported change in bowel habit. [f]In six of these cases patient had reported change in bowel habit. N/A: not applicable

Patient-reported symptom severity

Patients reported that most symptoms (except for change in bowel habit and change in bowel habit-diarrhoea) were usually either mild or moderate. “Severe” and “very severe” symptoms were reported for all types of change in bowel habit more frequently than for other symptoms (Figure 2).

The proportion of GPs recording symptoms often increased based on patient-reported symptom severity. This was particularly evident for rectal bleeding, abdominal pain and unexplained weight loss, although there was variation due to small numbers (particularly very few “very severe” or “unbearable” cases) (Figure 2).

[Insert Figure 2 here]

Clinical outcomes

Clinical outcomes were only available for 105 patients in the full dataset (34%); 26 had a positive FIT result (25%). Three patients received a CRC diagnosis (3% of 105 patients and 12% of FIT positives), comprising two rectal cancers and one carcinoma of the caecum. All CRC cases had positive FIT results with values >400 µg Hb/g faeces. One patient was diagnosed with endometrial cancer (negative FIT result). Twenty-two patients received an alternative gastrointestinal diagnosis, most commonly diverticular disease, diverticulosis or diverticulitis (n=12).

Discussion

This study set in routine English primary care showed that FIT usage was greater in females and in older patients - perhaps unsurprisingly as CRC incidence increases with age, but the gender balance is different than expected as colorectal cancer is more common in males.[1] Patients reported on more symptoms than GPs recorded when ordering FITs. While GP reporting of symptoms increased based on symptom severity, agreement between patient- and GP-reporting was low suggesting that risk assessment algorithms such as RATs [27] could under-perform. Pre-pandemic we aimed to understand real-life use of FIT for patients presenting in primary care with symptoms that could indicate CRC, particularly those with lower risk symptoms. As the COVID-19 pandemic progressed it became clear that FIT was

For peer review only - http://bmjopen.bmj.com/site/about/guidelines.xhtml

being used more extensively for patients with higher-risk symptoms, therefore we stopped the study prematurely.

This study provides much needed data on agreement between patient-reported and GP-recorded symptoms prompting a FIT request. To our knowledge, this is the first time that these findings are reported for English populations. While this study was neither designed nor powered to look at cancer outcomes, poor access to primary care records compounded this aspect, and was a key limitation. The analytic approach, creating a full dataset that contained key variables for all patients (symptom data and FIT results) reduced our sample size, with an over-representation of the least deprived and older patients. The study also over-represented the White British population (estimated to be 85.2% in the East of England according to the latest Census).[28] Nonetheless, comparisons with historical data from the whole of East of England showed similar patterns regarding the proportion of positive FITs, and on GP-recorded symptoms. Finally, patients did not provide information on symptoms at the same time as GPs (increasing the risk of recall bias), and different symptom collection methods were used.

Under-recording of symptoms by healthcare professionals is common and has also been previously linked to symptom severity and their perceived importance.[29-34] We identified only one other study describing both patient- and GP-reported symptoms when a FIT is requested in primary care.[34] Set in Swedish primary care, Högberg *et al* also described low symptom agreement, with GPs reporting symptoms less often[34]. In our study, GPs were compelled to tick one or more symptoms from a list to request a FIT; GPs may have chosen to record only the symptoms that triggered clinical action, and/or prioritised what to record. Their clinical interpretation of a symptom (such as its importance or duration) may also differ from the patient perspective. This may explain why GPs reported symptoms which sometimes were not described by patients (often alarm symptoms such as abdominal pain, rectal bleeding and change in bowel habit), and why agreement was highest for unexplained weight loss and rectal bleeding, both alarm symptoms. Some patients may have under-reported their symptoms to their GP, or only mentioned the most severe symptom. Nonetheless, low symptom agreement between GPs and patients suggests possible recording bias and sub-optimal record-keeping. These can have implications for patient management, such as the risk of underestimating the importance of certain symptoms for patients' cancer risk assessment and quality of life. This possibility needs to be further investigated.

The Swedish study found the most common patient-reported symptoms were abdominal pain and diarrhoea, in contrast to change in bowel habit and fatigue in our study[34]. Both studies

For peer review only - http://bmjopen.bmj.com/site/about/guidelines.xhtml

reported the same two most common GP-reported symptoms, abdominal pain and change in bowel habit. These symptoms, alongside age criteria, could potentially trigger an urgent referral in the UK.[4] It may be that GPs assessed these patients as being lower risk, based on other clinical characteristics.[11, 15] Our results suggest that FIT is being used to aid assessment of “grey area” patients, in line with its initially intended use. However, the high proportion of reported alarm symptoms, alongside older populations, also indicate that FIT was being used beyond its intended use, including for patients previously considered to be higher-risk. This was partly due to new guidance brought in response to the COVID-19 pandemic but cannot wholly be explained by this public health emergency.

Importantly, this wider adoption of FIT does not mean that the more recent use is not appropriate. Recent studies do show that FIT has the potential to be used more widely.[9, 10, 17] Perhaps FIT use has become more embedded and widespread in primary care, and clinicians have become more confident with its use(s). With access to hospital services including endoscopy under pressure during the pandemic, FIT has become a standard filter triage test in vague symptom pathways and is now an integral part of most 2WW colorectal pathways.[35, 36] Furthermore, recent guidance from the Association of Coloproctology of Great Britain & Ireland and the British Society of Gastroenterology recommends the use of FIT as a diagnostic triage tool in primary care.[37] There is also ongoing work being conducted to support the introduction of using FIT results to risk stratify patients into a 3-tier referral system for full colonoscopy, capsule endoscopy and no further investigation [38]. Future research will be required to assess this expanding role of FIT outside the current NICE referral guidelines.

Figure legends

Figure 1. Study flow diagram

[a]16 patients returned more than one questionnaire. The first response was kept to reduce recall bias
GP: general practitioner. FIT: faecal immunochemical test.

Figure 2. Symptom severity reported by patients

Supplementary data

Strobe checklist

Supplementary File 1. Patient questionnaire

Supplementary File 2. FIT requests and GP-recorded symptoms in the South East of England between November 2018 and May 2020

Supplementary Figure 1. Patient-reported symptom patterns

Supplementary Table 1. Patient characteristics according to availability of linked data

Additional information

For peer review only - http://bmjopen.bmj.com/site/about/guidelines.xhtml

Data sharing statement

Anonymised data are available from the authors upon reasonable request.

Funding

This research arises from the CanTest Collaborative, which is funded by Cancer Research UK C8640/A23385, of which Fiona M Walter and Willie Hamilton are Directors, Niek de Wit is an Associate Director, Natalia Calanzani and Merel M Pannebakker are postdoctoral researchers. The funders of the study had no role in study design, data collection, data analysis, data interpretation, or writing of the report.

For peer review only

Ethical approval

Ethical approval for the FIT-East study was granted by the East of England Cambridge Central Research Ethics Committee – reference 19/EE/0036.

Competing interests

None declared.

Patient and public involvement

There was no PPI involvement in the quantitative component of the study reported here.

Patient consent for publication

Not applicable.

Contributors

MMP and FMW planned the original study; WH and FMW secured funding. Analysis was planned by MMP, NC, MJT, HW, NdW, WH, PH and FMW and carried out by NC, MJT, HW and FMW. NC drafted the manuscript with senior input from WH, NdW and FMW and revised it after critical reviews from all authors. All authors read and approved the final manuscript.

Acknowledgements

We thank all the patients who agreed to take part in the study, and who completed the questionnaire. We thank the general practice staff who provided us with patient outcome data. We thank Allison Chipchase, Eve Calderbank and Claire Corbett for their help obtaining historical data. We thank the North East Essex and Suffolk Pathology Services at the East Suffolk and North Essex NHS Foundation Trust, and the Clinical Biochemistry section of Laboratory Medicine at Norfolk and Norwich University Hospitals NHS Foundation Trust for all their help providing laboratory data. We are grateful to Dr Marije van Melle for her work in the

For peer review only - http://bmjopen.bmj.com/site/about/guidelines.xhtml

early stages of study design, and to Dr Sarah ER Bailey for her help during protocol development. We are grateful to Dr Lina Massou for expert statistical input. We thank Aina Chang and Sara Shaida for their help with data collection and gathering background information for the study, James Brimicombe for data management advice, support and expertise. We are indebted to Andy Cowan for his invaluable support collecting and managing study data, and for his comments on the study results. We also thank Victoria Hardy, Dr Owain T Jones and Dr Yin Zhou for their comments on the study results.

References

1. GLOBOCAN 2018. Estimated number of deaths worldwide, both sexes, all ages 2020 [Available from: https://gco.iarc.fr/].
2. National Cancer Registration and Analysis Service (NCRAS). Percentage of Diagnoses by route - 2006 to 2016. In: (a) RtD-w, editor. Microsoft Excel2019.
3. Koo MM, von Wagner C, Abel GA, McPhail S, Hamilton W, Rubin GP, et al. The nature and frequency of abdominal symptoms in cancer patients and their associations with time to help-seeking: evidence from a national audit of cancer diagnosis. Journal of Public Health. 2018;40(3):e388-e95.
4. NICE. Suspected cancer: recognition and referral NICE guideline [NG12] 2015 [Available from: https://www.nice.org.uk/guidance/ng12].
5. Hamilton W. The CAPER studies: five case-control studies aimed at identifying and quantifying the risk of cancer in symptomatic primary care patients. British journal of cancer. 2009;101 Suppl 2(Suppl 2):S80-S6.
6. NICE. Quantitative faecal immunochemical tests to guide referral for colorectal cancer in primary care (DG30) 2017 [Available from: https://www.nice.org.uk/guidance/dg30].
7. Westwood M, Corro Ramos I, Lang S, Luyendijk M, Zaim R, Stirk L, et al. Faecal immunochemical tests to triage patients with lower abdominal symptoms for suspected colorectal cancer referrals in primary care: a systematic review and cost-effectiveness analysis. Health Technol Assess. 2017;21(33):1-234.
8. Walter FM, Thompson MJ, Wellwood I, Abel GA, Hamilton W, Johnson M, et al. Evaluating diagnostic strategies for early detection of cancer: the CanTest framework. BMC Cancer. 2019;19(1):586.
9. Bailey SER, Abel GA, Atkins A, Byford R, Davies SJ, Mays J, et al. Diagnostic performance of a faecal immunochemical test for patients with low-risk symptoms of colorectal cancer in primary care: an evaluation in the South West of England. Br J Cancer. 2021.
10. Digby J, Strachan JA, McCann R, Steele RJ, Fraser CG, Mowat C. Measurement of faecal haemoglobin with a faecal immunochemical test can assist in defining which patients attending primary care with rectal bleeding require urgent referral. Annals of Clinical Biochemistry. 2020;57(4):325-7.
11. Nicholson BD, James T, Paddon M, Justice S, Oke JL, East JE, et al. Faecal immunochemical testing for adults with symptoms of colorectal cancer attending English primary care: a retrospective cohort study of 14 487 consecutive test requests. Alimentary Pharmacology & Therapeutics. 2020;17:17.
12. Chapman C, Thomas C, Morling J, Tangri A, Oliver S, Simpson JA, et al. Early clinical outcomes of a rapid colorectal cancer diagnosis pathway using faecal immunochemical testing in Nottingham. Colorectal Disease. 2020;22(6):679-88.
13. Hogberg C, Gunnarsson U, Cronberg O, Thulesius H, Lilja M, Jansson S. Qualitative faecal immunochemical tests (FITs) for diagnosing colorectal cancer in patients with histories of rectal bleeding in primary care: a cohort study. International Journal of Colorectal Disease. 2020;35(11):2035-40.

For peer review only - http://bmjopen.bmj.com/site/about/guidelines.xhtml

14. Hogberg C, Gunnarsson U, Jansson S, Thulesius H, Cronberg O, Lilja M. Diagnosing colorectal cancer in primary care: cohort study in Sweden of qualitative faecal immunochemical tests, haemoglobin levels, and platelet counts. British Journal of General Practice. 2020;70(701):e843-e51.
15. Juul JS, Hornung N, Andersen B, Laurberg S, Olesen F, Vedsted P. The value of using the faecal immunochemical test in general practice on patients presenting with non-alarm symptoms of colorectal cancer. British Journal of Cancer. 2018;119(4):471-9.
16. Mowat C, Digby J, Strachan JA, McCann R, Hall C, Heather D, et al. Impact of introducing a faecal immunochemical test (FIT) for haemoglobin into primary care on the outcome of patients with new bowel symptoms: a prospective cohort study. BMJ Open Gastroenterology. 2019;6(1):e000293.
17. Pin-Vieito N, Tejido-Sandoval C, de Vicente-Bielza N, Sánchez-Gómez C, Cubiella J. Faecal immunochemical tests safely enhance rational use of resources during the assessment of suspected symptomatic colorectal cancer in primary care: systematic review and meta-analysis. Gut. 2021:gutjnl-2021-324856.
18. Loveday C, Sud A, Jones ME, Broggio J, Scott S, Gronthound F, et al. Prioritisation by FIT to mitigate the impact of delays in the 2-week wait colorectal cancer referral pathway during the COVID-19 pandemic: a UK modelling study. Gut. 2020;27:27.
19. Office for National Statistics. Table SAPE22DT6a: Mid-2019 Population Estimates for 2020 Clinical Commissioning Groups in England by Single Year of Age and Sex, Persons - National Statistics 2020 [Available from: https://www.ons.gov.uk/peoplepopulationandcommunity/populationandmigration/populationestimates/datasets/clinicalcommissioninggroupmidyearpopulationestimates.
20. Walter FM, Emery JD, Mendonca S, Hall N, Morris HC, Mills K, et al. Symptoms and patient factors associated with longer time to diagnosis for colorectal cancer: results from a prospective cohort study. Br J Cancer. 2016;115(5):533-41.
21. Walter FM, Mills K, Mendonça SC, Abel GA, Basu B, Carroll N, et al. Symptoms and patient factors associated with diagnostic intervals for pancreatic cancer (SYMPTOM pancreatic study): a prospective cohort study. Lancet Gastroenterol Hepatol. 2016;1(4):298-306.
22. Walter FM, Rubin G, Bankhead C, Morris HC, Hall N, Mills K, et al. Symptoms and other factors associated with time to diagnosis and stage of lung cancer: a prospective cohort study. Br J Cancer. 2015;112 Suppl 1(Suppl 1):S6-13.
23. Emery JD, Reid G, Prevost AT, Ravine D, Walter FM. Development and validation of a family history screening questionnaire in Australian primary care. Ann Fam Med. 2014;12(3):241-9.
24. Walter FM, Prevost AT, Birt L, Grehan N, Restarick K, Morris HC, et al. Development and evaluation of a brief self-completed family history screening tool for common chronic disease prevention in primary care. Br J Gen Pract. 2013;63(611):e393-400.
25. Landis JR, Koch GG. The measurement of observer agreement for categorical data. Biometrics. 1977;33(1):159-74.
26. Sultan M, Abebe Y, Tsadik AW, Ababa A, Yesus AG, Mould-Millman N-K. Trends and barriers of emergency medical service use in Addis Ababa; Ethiopia. BMC emergency medicine. 2019;19(1):28.
27. ERICA TRIAL. https://www.theericatrial.co.uk/2022 [
28. Office for National Statistics. England and Wales 2011 Census. Population by ethnicity and region 2018 [Available from: https://www.ethnicity-facts-figures.service.gov.uk/uk-population-by-ethnicity/national-and-regional-populations/regional-ethnic-diversity/latest/downloads/population-by-ethnicity-and-region.csv.
29. Chidambaram S, DeShields T, Potter P, Olsen S, Chen L. Patient and provider concordance on symptoms during the oncology outpatient clinic visit. J Community Support Oncol. 2014;12(10):370-7.
30. Valikodath NG, Newman-Casey PA, Lee PP, Musch DC, Niziol LM, Woodward MA. Agreement of Ocular Symptom Reporting Between Patient-Reported Outcomes and Medical Records. JAMA Ophthalmol. 2017;135(3):225-31.

For peer review only - http://bmjopen.bmj.com/site/about/guidelines.xhtml

31. Horvath Walsh LE, Rider A, Piercy J, Pike J, Wilson S, Pandya BJ, et al. Real-World Impact of Physician and Patient Discordance on Health-Related Quality of Life in US Patients with Acute Myeloid Leukemia. Oncol Ther. 2019;7(1):67-81.
32. Strömgren AS, Groenvold M, Pedersen L, Olsen AK, Spile M, Sjøgren P. Does the medical record cover the symptoms experienced by cancer patients receiving palliative care? A comparison of the record and patient self-rating. J Pain Symptom Manage. 2001;21(3):189-96.
33. Pakhomov SV, Jacobsen SJ, Chute CG, Roger VL. Agreement between patient-reported symptoms and their documentation in the medical record. Am J Manag Care. 2008;14(8):530-9.
34. Högberg C, Karling P, Rutegård J, Lilja M. Patient-reported and doctor-reported symptoms when faecal immunochemical tests are requested in primary care in the diagnosis of colorectal cancer and inflammatory bowel disease: a prospective study. BMC Family Practice. 2020;21(1):129.
35. NHS England. Clinical guide for triaging patients with lower gastrointestinal symptoms 2020 [Available from: https://www.nice.org.uk/Media/Default/About/COVID-19/Specialty-guides/triaging-patients-with-lower-gi-symptoms.pdf (accessed 20 Jul 2021).
36. Turvill JL, Turnock D, Cottingham D, Haritakis M, Jeffery L, Girdwood A, et al. The Fast Track FIT study: diagnostic accuracy of faecal immunochemical test for haemoglobin in patients with suspected colorectal cancer. British Journal of General Practice. 2021:BJGP.2020.1098.
37. Guideline Development Group (GDG). Faecal Immunochemical Testing (FIT) in patients with signs or symptoms of suspected colorectal cancer (CRC): A joint guideline from the Association of Coloproctology of Great Britain & Ireland (ACPGBI) and the British Society of Gastroenterology (BSG) https://www.bsg.org.uk/clinical-resource/faecal-immunochemical-testing-fit-in-patients-with-signs-or-symptoms-of-suspected-colorectal-cancer-crc-a-joint-guideline-from-the-acpgbi-and-the-bsg/: Association of Coloproctology of Great Britain & Ireland (ACPGBI) and the British Society of Gastroenterology (BSG); 2022 [
38. Norfolk and Norwich University Hospitals. Guideline for the Triage and Diagnostic Investigation of Patients Referred with Suspected Colorectal Cancer www.nnuh.nhs.uk2020 [

For peer review only - http://bmjopen.bmj.com/site/about/guidelines.xhtml

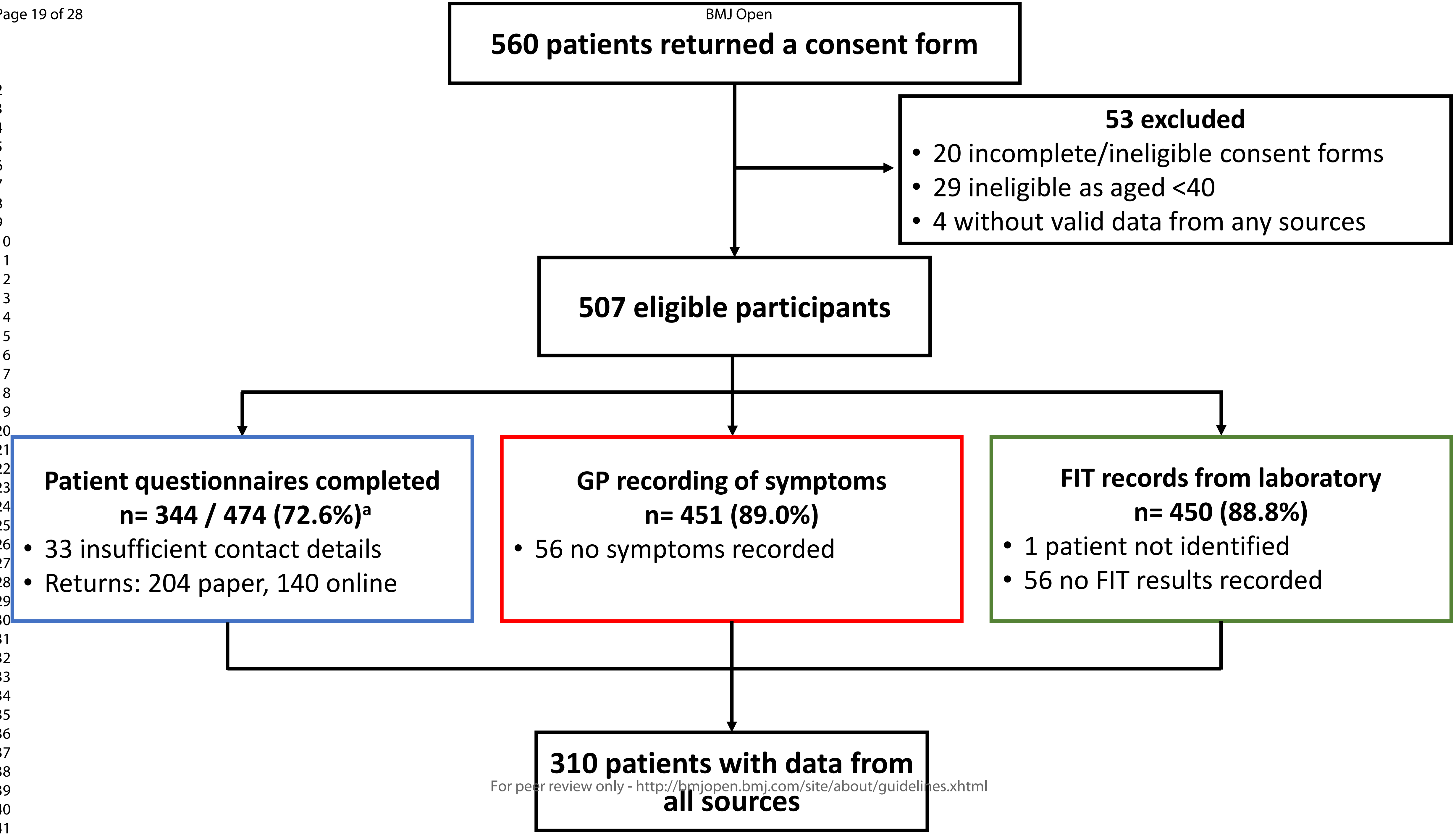
560 patients returned a consent form
53 excluded
• 20 incomplete/ineligible consent forms
• 29 ineligible as aged <40
• 4 without valid data from any sources
507 eligible participants
Patient questionnaires completed
n= 344 / 474 (72.6%)[a]
• 33 insufficient contact details
• Returns: 204 paper, 140 online
GP recording of symptoms
n= 451 (89.0%)
• 56 no symptoms recorded
FIT records from laboratory
n= 450 (88.8%)
• 1 patient not identified
• 56 no FIT results recorded
310 patients with data from
all sources

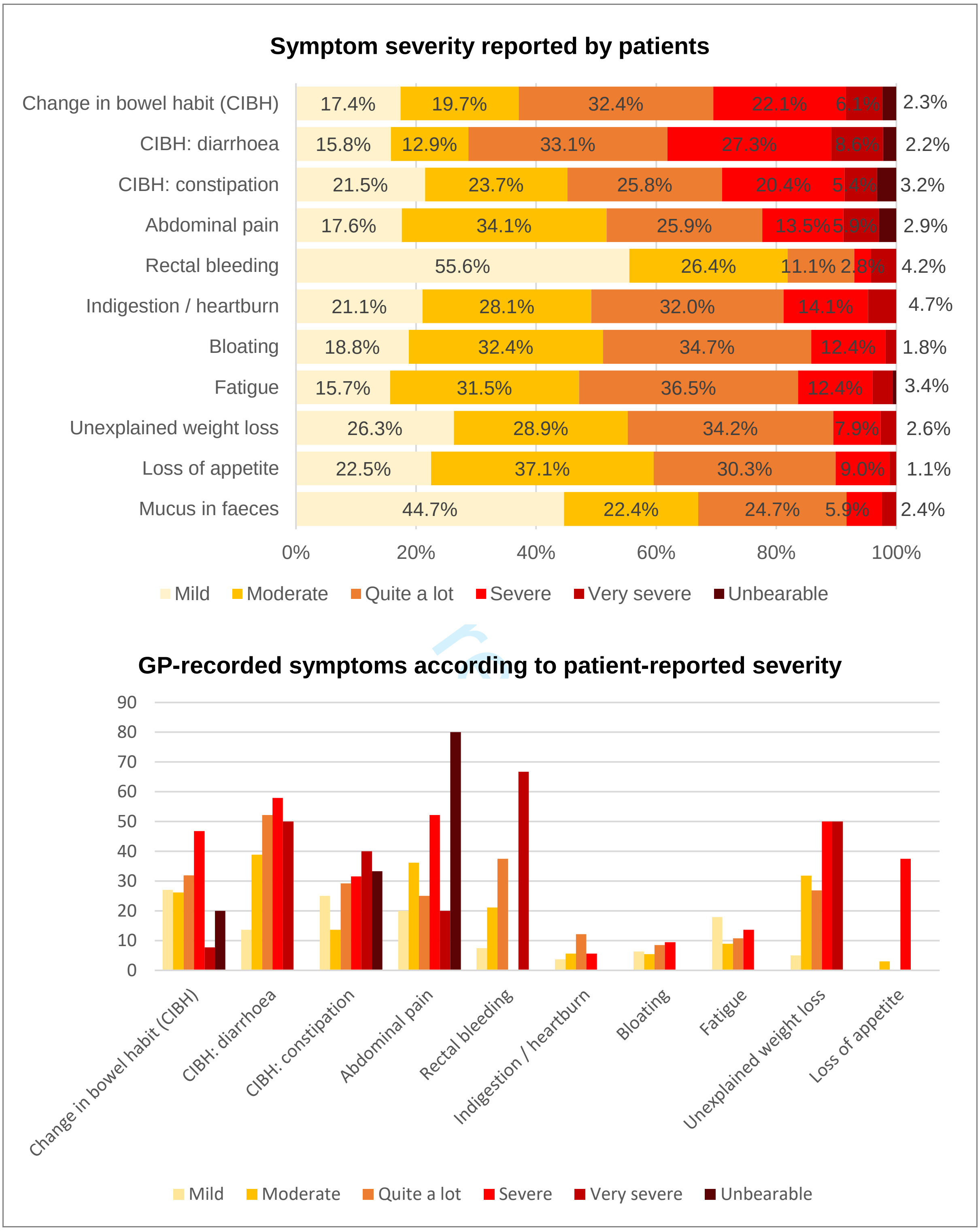
Symptom severity reported by patients
Change in bowel habit (CIBH) 17.4% 19.7% 32.4% 22.1% 6.1% 2.3%
CIBH: diarrhoea 15.8% 12.9% 33.1% 27.3% 8.6% 2.2%
CIBH: constipation 21.5% 23.7% 25.8% 20.4% 5.4% 3.2%
Abdominal pain 17.6% 34.1% 25.9% 13.5% 5.9% 2.9%
Rectal bleeding 55.6% 26.4% 11.1% 2.8% 4.2%
Indigestion / heartburn 21.1% 28.1% 32.0% 14.1% 4.7%
Bloating 18.8% 32.4% 34.7% 12.4% 1.8%
Fatigue 15.7% 31.5% 36.5% 12.4% 3.4%
Unexplained weight loss 26.3% 28.9% 34.2% 7.9% 2.6%
Loss of appetite 22.5% 37.1% 30.3% 9.0% 1.1%
Mucus in faeces 44.7% 22.4% 24.7% 5.9% 2.4%
0% 20% 40% 60% 80% 100%
Mild Moderate Quite a lot Severe Very severe Unbearable
GP-recorded symptoms according to patient-reported severity
0 10 20 30 40 50 60 70 80 90
Change in bowel habit (CIBH)
CIBH: diarrhoea
CIBH: constipation
Abdominal pain
Rectal bleeding
Indigestion / heartburn
Bloating
Fatigue
Unexplained weight loss
Loss of appetite
Mild Moderate Quite a lot Severe Very severe Unbearable

Supplementary data

Who are the patients being offered the faecal immunochemical test in routine English general practice? A prospective descriptive study

Supplementary File 1. Patient questionnaire

Section 1: About Your Symptoms
We are interested in symptoms which you think are related to your recent poo test request. Did you have any of the following symptoms or health problems?

1. Change in bowel habit
☐ Yes ☐ No If YES please indicate the severity:
☐ Mild ☐ Moderate ☐ Quite a lot ☐ Severe ☐ Very severe ☐ Unbearable

2. Change in bowel habit – mainly diarrhoea or loose poo
☐ Yes ☐ No If YES please indicate the severity:
☐ Mild ☐ Moderate ☐ Quite a lot ☐ Severe ☐ Very severe ☐ Unbearable

3. Change in bowel habit – mainly constipation
☐ Yes ☐ No If YES please indicate the severity:
☐ Mild ☐ Moderate ☐ Quite a lot ☐ Severe ☐ Very severe ☐ Unbearable

4. Abdominal pain or discomfort that wasn't normal for you
☐ Yes ☐ No If YES please indicate the severity:
☐ Mild ☐ Moderate ☐ Quite a lot ☐ Severe ☐ Very severe ☐ Unbearable

5. Bleeding from your back passage
☐ Yes ☐ No If YES please indicate the severity:
☐ Mild ☐ Moderate ☐ Quite a lot ☐ Severe ☐ Very severe ☐ Unbearable

6. Indigestion or heartburn
☐ Yes ☐ No If YES please indicate the severity:
☐ Mild ☐ Moderate ☐ Quite a lot ☐ Severe ☐ Very severe ☐ Unbearable

7. Bloating
☐ Yes ☐ No If YES please indicate the severity:
☐ Mild ☐ Moderate ☐ Quite a lot ☐ Severe ☐ Very severe ☐ Unbearable

8. Fatigue or tiredness that is unusual for you
☐ Yes ☐ No If YES please indicate the severity:
☐ Mild ☐ Moderate ☐ Quite a lot ☐ Severe ☐ Very severe ☐ Unbearable

9. Unexplained weight loss
☐ Yes ☐ No If YES please indicate the severity:
☐ Mild ☐ Moderate ☐ Quite a lot ☐ Severe ☐ Very severe ☐ Unbearable

10. Loss of appetite
☐ Yes ☐ No If YES please indicate the severity:
☐ Mild ☐ Moderate ☐ Quite a lot ☐ Severe ☐ Very severe ☐ Unbearable

11. Mucous or slime in poo
☐ Yes ☐ No If YES please indicate the severity:
☐ Mild ☐ Moderate ☐ Quite a lot ☐ Severe ☐ Very severe ☐ Unbearable

Section 2: About You
12. How would you describe your ethnicity? Please tick one box only
White

For peer review only

☐ British ☐ Irish
☐ Any other white background. Please write...

Mixed
☐ White and Black Caribbean ☐ White and Black African ☐ White and Asian
☐ Any other Mixed background. Please write...

Chinese or other ethnic group
☐ Chinese ☐ Any other ethnic group. Please write...

Asian or Asian British
☐ Indian ☐ Pakistani ☐ Bangladeshi ☐ Any other Asian background. Please write...............................

Black or Black British
☐ Caribbean ☐ African ☐ Any other Black background. Please write...

13. What is your highest level of qualification? Please tick one box only
☐ Degree (or equivalent) ☐ Other higher education ☐ A-Level (or equivalent)
☐ GCSE/O-Level (or equivalent) ☐ None ☐ Other, please specify................................

14. Do you use any of the following medications?
☐ NSAID/Anti-inflammatory painkillers ☐ Aspirin
☐ Anticoagulants/blood thinners (e.g. warfarin, coumarines) ☐ Antidepressants

15. Are you suffering from, or have you suffered from, any of the following? Please tick any that are applicable to you
☐ Inflammatory bowel disease (e.g. Crohn's or colitis) ☐ Diabetes o Irritable bowel syndrome
☐ Arthritis ☐ Peptic ulcer ☐ Anxiety or depression o Bowel (colon or rectal) cancer ☐ Bowel polyps
☐ Other cancer. Please write which type..

16. Have you had a colonoscopy, sigmoidoscopy or bowel scope?
In the past 5 years? ☐ Yes ☐ No
Ever? ☐ Yes ☐ No

17. Have you had a bowel screening test or poo test?
In the past 5 years? ☐ Yes ☐ No
Ever? ☐ Yes ☐ No

18. Smoking - please tick one box only. Are you?
☐ A current smoker? ☐ An ex-smoker? ☐ A non-smoker (never smoked)?

19. Alcohol. How often have you had an alcoholic drink of any kind in the last 12 months? Please tick one
☐ Almost every day ☐ About once a week ☐ About twice a week ☐ About once a fortnight
☐ Only a few times a year ☐ I never drink alcohol

20. What is your height (fill in one of the options)?
In Feet and Inches or CM..

21. What is your current weight (fill in one of the options)?
Stones and pounds..Kg..

22. Have any of your close relatives, including parent(s), children, brother(s) or sister(s), had colon or rectal cancer (also known as large bowel or colorectal cancer) before the age of 55?
☐ Yes ☐ No

23. Do you have more than one relative who has had colon or rectal cancer on either your mother's or father's side of the family? Please think about all of the following relatives: parent(s), children, brother(s), sister(s), grandparent(s), aunt(s), uncle(s), niece(s), nephew(s) and grandchildren.
☐ Yes ☐ No

For peer review only

Supplementary File 2. FIT requests and GP-recorded symptoms in the South East of England between November 2018 and May 2020

Anonymised data on FIT requests, FIT results and GP-recorded symptom/s with FIT request covering the period from FIT implementation in November 2018 to May 2020.

From November 2018 to May 2020, 15,620 valid FITs were performed in this region. The number of FITs ordered increased from November 2018 to May 2020, reaching a peak in January 2020 (1512 valid tests), reducing during the early pandemic (compared with January, March, 26.3% and April, 50.1% decreases respectively), and increasing again in May 2020 (1465 valid tests) (Figure 1). Of the 15,620 valid FITs performed during this period, 20.7% (n=3237) were positive (range 15.1% (November 2018, Norfolk only) to 29.1% (both areas, April 2020)).

Figure 1. All FIT requests and FIT positives in Eastern England post DG30 implementation

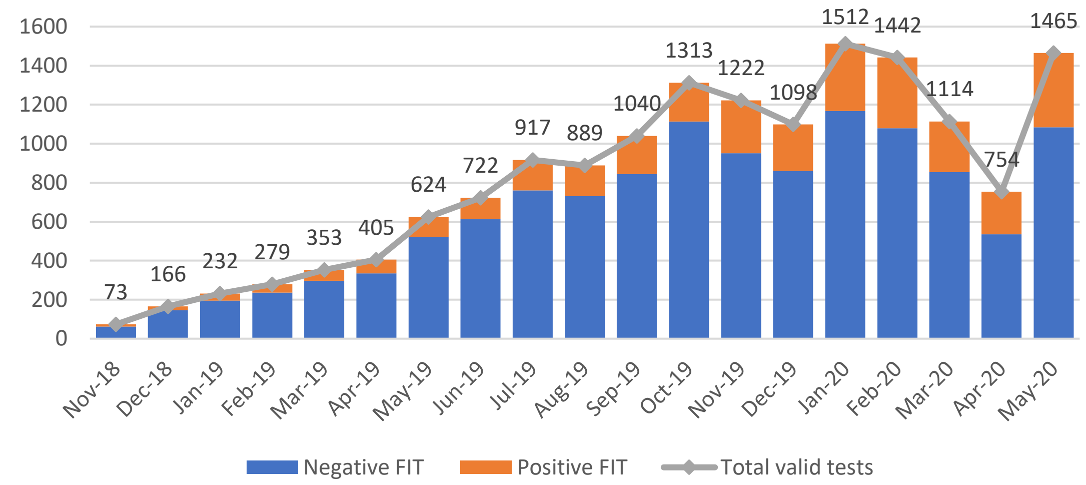

The proportion of GP-recorded symptoms was lower when compared to patient-reported symptoms, and similar to GP-recorded symptoms in the study. This was the case for all reported symptoms (Figure 2).

Figure 2. Historical and FIT-East data for symptom reporting

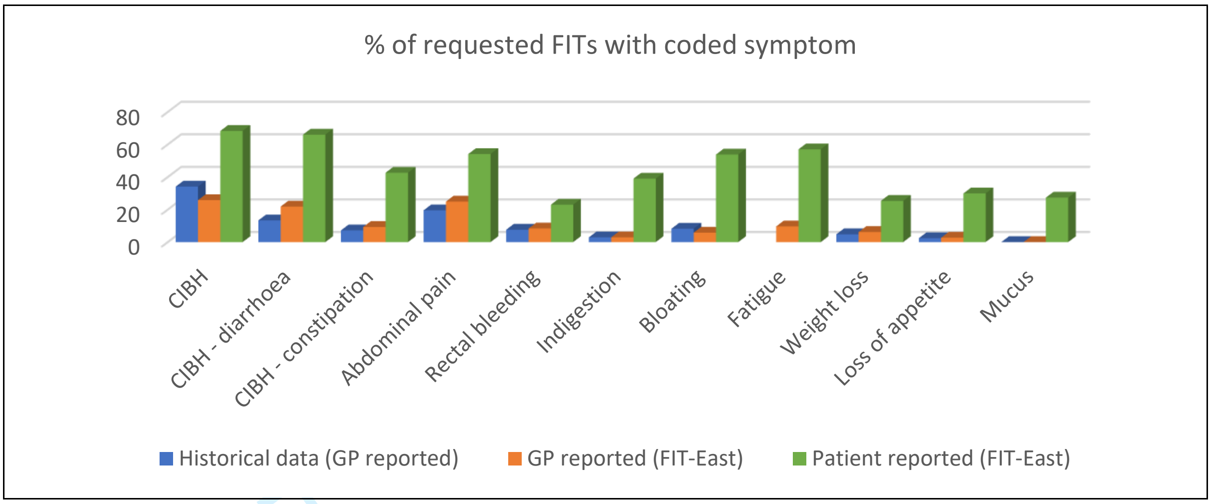

Historical, aggregated data has no information on fatigue, and mucus was only available as an answer option in the patient questionnaire.

For peer review only

Supplementary Table 1. Patient characteristics according to availability of linked data

Characteristics	Linked data available (full dataset) (n=310)	Linked data not available (n=197)	Tests
Age			
Median age (IQR)	70 (61-77)	66 (53.5-75.0)	**U=36222.5, *p*<0.001**
Gender	n (%)	n (%)	
Female	160 (53)	100 (52)	
Male	142 (47)	93 (48)	χ(1)=0.064, *p*=0.80
Deprivation (IMD Quintiles)	n (%)	n (%)	
1 (least deprived)	69 (22)	30 (18)	**U=22457, *p*=0.04**
2	74 (24)	26 (16)	
3	88 (28)	55 (34)	
4	49 (16)	34 (21)	
5 (most deprived)	30 (10)	18 (11)	

For peer review only

Supplementary Figure 1. Patient-reported symptom patterns

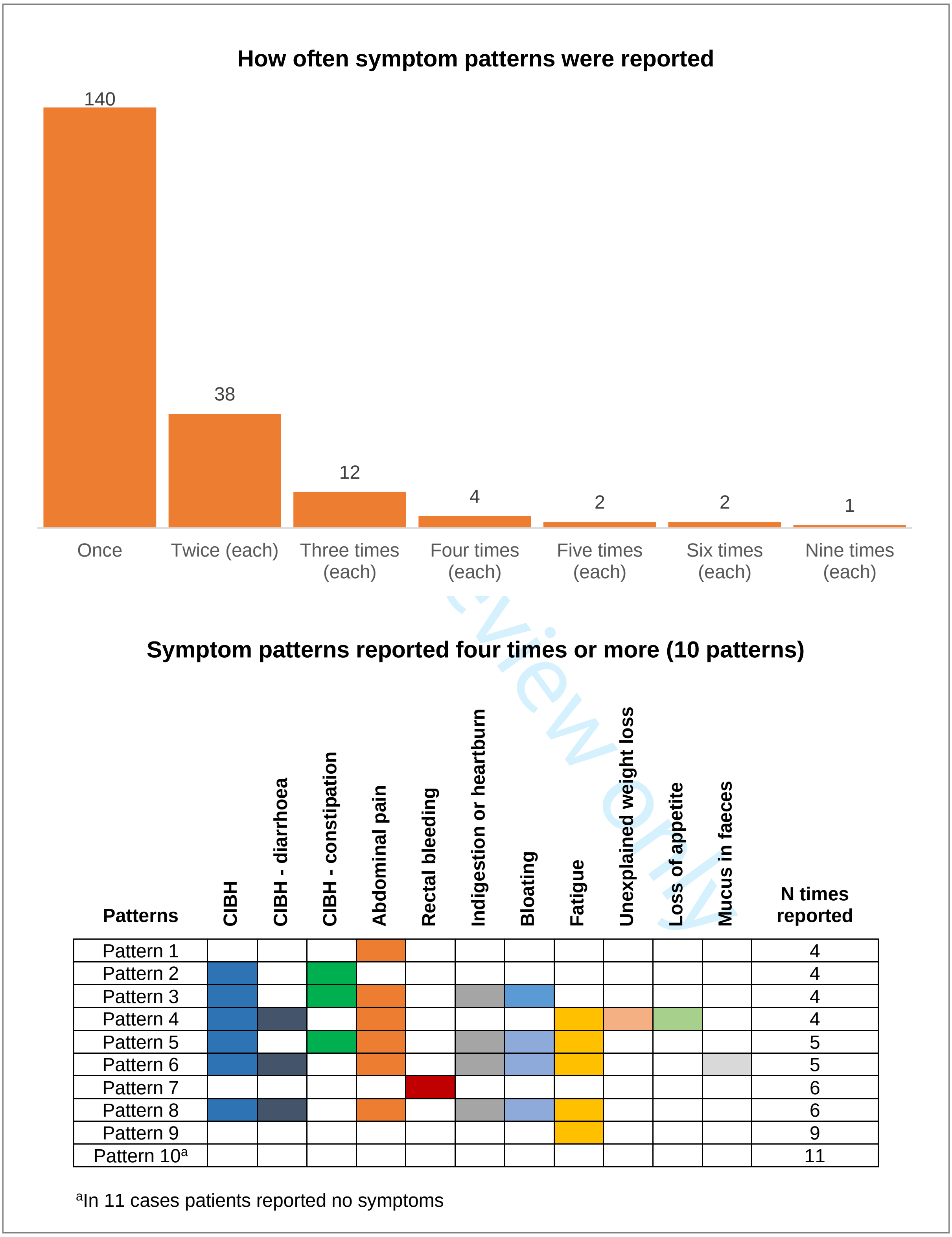

STROBE Statement—checklist of items that should be included in reports of observational studies

	Item No	Recommendation	Page No
**Title and abstract**	1	(*a*) Indicate the study's design with a commonly used term in the title or the abstract	1
		(*b*) Provide in the abstract an informative and balanced summary of what was done and what was found	2
**Introduction**			
Background/rationale	2	Explain the scientific background and rationale for the investigation being reported	4
Objectives	3	State specific objectives, including any prespecified hypotheses	4
**Methods**			
Study design	4	Present key elements of study design early in the paper	4
Setting	5	Describe the setting, locations, and relevant dates, including periods of recruitment, exposure, follow-up, and data collection	5
Participants	6	(*a*) *Cohort study*—Give the eligibility criteria, and the sources and methods of selection of participants. Describe methods of follow-up<br>*Case-control study*—Give the eligibility criteria, and the sources and methods of case ascertainment and control selection. Give the rationale for the choice of cases and controls<br>*Cross-sectional study*—Give the eligibility criteria, and the sources and methods of selection of participants	5-6
		(*b*) *Cohort study*—For matched studies, give matching criteria and number of exposed and unexposed<br>*Case-control study*—For matched studies, give matching criteria and the number of controls per case	N/A
Variables	7	Clearly define all outcomes, exposures, predictors, potential confounders, and effect modifiers. Give diagnostic criteria, if applicable	6-7
Data sources/ measurement	8*	For each variable of interest, give sources of data and details of methods of assessment (measurement). Describe comparability of assessment methods if there is more than one group	5-7, footnote in Table 2
Bias	9	Describe any efforts to address potential sources of bias	Page 6, choice of methods for outcome data
Study size	10	Explain how the study size was arrived at	As above; this was an exploratory descriptive study
Quantitative variables	11	Explain how quantitative variables were handled in the analyses. If applicable, describe which groupings were chosen and why	6-7
Statistical methods	12	(*a*) Describe all statistical methods, including those used to control for confounding	6-7
		(*b*) Describe any methods used to examine subgroups and interactions	6-7
		(*c*) Explain how missing data were addressed	6-7
		(*d*) *Cohort study*—If applicable, explain how loss to follow-up was addressed<br>*Case-control study*—If applicable, explain how matching of cases and controls was addressed	N/A

For peer review only - http://bmjopen.bmj.com/site/about/guidelines.xhtml

		Cross-sectional study—If applicable, describe analytical methods taking account of sampling strategy	
		(*e*) Describe any sensitivity analyses	N/A
**Results**			
Participants	13*	(a) Report numbers of individuals at each stage of study—eg numbers potentially eligible, examined for eligibility, confirmed eligible, included in the study, completing follow-up, and analysed	7, Figure 1
		(b) Give reasons for non-participation at each stage	7, Figure 1
		(c) Consider use of a flow diagram	Figure 1
Descriptive data	14*	(a) Give characteristics of study participants (eg demographic, clinical, social) and information on exposures and potential confounders	7-11, Tables 1-3, Figure 2
		(b) Indicate number of participants with missing data for each variable of interest	Footnote in Tables 1 and 2, "clinical outcomes" subsection (p.11)
		(c) *Cohort study*—Summarise follow-up time (eg, average and total amount)	N/A
Outcome data	15*	*Cohort study*—Report numbers of outcome events or summary measures over time	*N/A*
		Case-control study—Report numbers in each exposure category, or summary measures of exposure	*N/A*
		Cross-sectional study—Report numbers of outcome events or summary measures	7-11, "clinical outcomes" subsection (p.11)
Main results	16	(*a*) Give unadjusted estimates and, if applicable, confounder-adjusted estimates and their precision (eg, 95% confidence interval). Make clear which confounders were adjusted for and why they were included	N/A
		(*b*) Report category boundaries when continuous variables were categorized	Footnote in Table 2
		(*c*) If relevant, consider translating estimates of relative risk into absolute risk for a meaningful time period	N/A
Other analyses	17	Report other analyses done—eg analyses of subgroups and interactions, and sensitivity analyses	N/A
**Discussion**			
Key results	18	Summarise key results with reference to study objectives	11
Limitations	19	Discuss limitations of the study, taking into account sources of potential bias or imprecision. Discuss both direction and magnitude of any potential bias	12
Interpretation	20	Give a cautious overall interpretation of results considering objectives, limitations, multiplicity of analyses, results from similar studies, and other relevant evidence	12-13
Generalisability	21	Discuss the generalisability (external validity) of the study results	12-13
**Other Information**			
Funding	22	Give the source of funding and the role of the funders for the present study and, if applicable, for the original study on which the present article is based	Funding statement, pages 13-14

*Give information separately for cases and controls in case-control studies and, if applicable, for exposed and unexposed groups in cohort and cross-sectional studies.

For peer review only

For peer review only - http://bmjopen.bmj.com/site/about/guidelines.xhtml

Note: An Explanation and Elaboration article discusses each checklist item and gives methodological background and published examples of transparent reporting. The STROBE checklist is best used in conjunction with this article (freely available on the Web sites of PLoS Medicine at http://www.plosmedicine.org/, Annals of Internal Medicine at http://www.annals.org/, and Epidemiology at http://www.epidem.com/). Information on the STROBE Initiative is available at www.strobe-statement.org.

For peer review only

BMJ Open

Who are the patients being offered the faecal immunochemical test in routine English general practice, and for what symptoms? A prospective descriptive study

Journal:	*BMJ Open*
Manuscript ID	bmjopen-2022-066051.R1
Article Type:	Original research
Date Submitted by the Author:	10-Aug-2022
Complete List of Authors:	Calanzani, Natalia ; University of Cambridge, Department of Public Health and Primary Care<br>Pannebakker, Merel M; University of Cambridge, Department of Public Health and Primary Care<br>Tagg, Max J; University of Cambridge, School of Clinical Medicine<br>Walford, Hugo; University College London, School of Clinical Medicine<br>Holloway, Peter; East of England Cancer Alliances<br>de Wit, Niek; Utrecht University, Julius Center for Health Sciences and Primary Care, University Medical Center<br>Hamilton, Willie; University of Exeter Medical School<br>Walter, Fiona M; Queen Mary University of London, Wolfson Institute of Population Health; University of Cambridge, Department of Public Health and Primary Care
**Primary Subject Heading**:	General practice / Family practice
Secondary Subject Heading:	Oncology, Health services research
Keywords:	Adult oncology < ONCOLOGY, Gastrointestinal tumours < ONCOLOGY, PRIMARY CARE

SCHOLARONE™
Manuscripts

I, the Submitting Author has the right to grant and does grant on behalf of all authors of the Work (as defined in the below author licence), an exclusive licence and/or a non-exclusive licence for contributions from authors who are: i) UK Crown employees; ii) where BMJ has agreed a CC-BY licence shall apply, and/or iii) in accordance with the terms applicable for US Federal Government officers or employees acting as part of their official duties; on a worldwide, perpetual, irrevocable, royalty-free basis to BMJ Publishing Group Ltd ("BMJ") its licensees and where the relevant Journal is co-owned by BMJ to the co-owners of the Journal, to publish the Work in this journal and any other BMJ products and to exploit all rights, as set out in our licence.

The Submitting Author accepts and understands that any supply made under these terms is made by BMJ to the Submitting Author unless you are acting as an employee on behalf of your employer or a postgraduate student of an affiliated institution which is paying any applicable article publishing charge ("APC") for Open Access articles. Where the Submitting Author wishes to make the Work available on an Open Access basis (and intends to pay the relevant APC), the terms of reuse of such Open Access shall be governed by a Creative Commons licence – details of these licences and which Creative Commons licence will apply to this Work are set out in our licence referred to above.

Other than as permitted in any relevant BMJ Author's Self Archiving Policies, I confirm this Work has not been accepted for publication elsewhere, is not being considered for publication elsewhere and does not duplicate material already published. I confirm all authors consent to publication of this Work and authorise the granting of this licence.

For peer review only

Who are the patients being offered the faecal immunochemical test in routine English general practice, and for what symptoms? A prospective descriptive study

Natalia Calanzani[1], Merel M Pannebakker[1], Max J Tagg[2], Hugo Walford[3], Peter Holloway[4], Niek de Wit[5], Willie Hamilton[6], Fiona M Walter[7,1,8]

[1]The Primary Care Unit, Department of Public Health and Primary Care, University of Cambridge, Cambridge, UK, ORCID ID: 0000-0002-5068-2543 (NC)
[2]School of Clinical Medicine, Addenbrooke's Hospital, University of Cambridge, UK, ORCID ID: 0000-0003-0341-3284
[3]School of Clinical Medicine, University College London, UK, ORCID ID: 0000-0001-9655-9674
[4]East of England Cancer Alliances, UK
[5]Julius Center for Health Sciences and Primary Care, University Medical Center, Utrecht University, Netherlands
[6]University of Exeter Medical School, Exeter, UK, ORCID ID: 0000-0003-1611-1373
[7]Wolfson Institute of Population Health, Queen Mary University of London, UK, ORCID ID: 0000-0002-7191-6476
[8]Centre for Cancer Research and Department of General Practice, University of Melbourne, Melbourne, Australia

Corresponding author:
Dr Natalia Calanzani, nm719@medschl.cam.ac.uk
The Primary Care Unit, Department of Public Health and Primary Care
Strangeways Research Laboratory, 2 Worts Causeway
University of Cambridge
Cambridge, CB1 8RN

Word count: 3134

For peer review only - http://bmjopen.bmj.com/site/about/guidelines.xhtml

Abstract

Objectives: The faecal immunochemical test (FIT) was introduced to triage patients with lower-risk symptoms of colorectal cancer (CRC) in English primary care in 2018. While there is growing evidence on its utility to triage patients in this setting, evidence is still limited on how official FIT guidance is being used, for which patients, and for what symptoms. We aimed to investigate the use of FIT in primary care practice for lower-risk patients who did not immediately meet criteria for urgent referral.

Design: A prospective, descriptive study of symptomatic patients offered a FIT in primary care between January and June 2020.

Setting: East of England general practices.

Participants: Consenting patients (aged ≥40 years) who were seen by their general practitioners (GPs) with symptoms of possible CRC for whom a FIT was requested. We excluded patients receiving a FIT for asymptomatic screening purposes, or patients deemed by GPs as lacking capacity for informed consent. Data were obtained via patient questionnaire, medical and laboratory records.

Primary and secondary outcome measures: FIT results (10 µg Hb/g faeces defined a positive result); patient sociodemographic and clinical characteristics; patient-reported and GP-recorded symptoms, symptom severity and symptom agreement between patient and GP (% and kappa statistics).

Results: Complete data were available for 310 patients, median age 70 (IQR 61-77), 53% female, and 23% FIT positive. Patients most commonly reported change in bowel habit (69%) and fatigue (57%) while GPs most commonly recorded abdominal pain (25%) and change in bowel habit (24%). Symptom agreement ranged from 44% (fatigue) to 80% (unexplained weight loss). Kappa agreement was universally low across symptoms.

Conclusion: Almost a quarter of this primary care cohort of symptomatic patients with FIT testing were found to be positive. However, there was low agreement between patient-reported and GP-recorded symptoms. This may impact cancer risk assessment and optimal patient management in primary care.

For peer review only - http://bmjopen.bmj.com/site/about/guidelines.xhtml

Keywords

Primary care, gastrointestinal tumours, adult oncology, adult gastroenterology

Strengths and limitations of this study

- Recent data on how FIT is used in primary care, and which symptoms trigger a FIT request
- To our knowledge, this is the first study in England to report on the agreement between patient-reported and GP-recorded symptoms
- Limitations include poor access to primary care records, and consequently limited evidence on cancer diagnoses
- There was over-representation of White British populations; therefore results may not be generalisable to different groups

For peer review only

Introduction

Colorectal cancer (CRC) is the second most common cause of cancer deaths worldwide and in the UK. [1] Despite screening programmes, most CRCs are diagnosed after symptomatic presentation, [2] with patients usually presenting first in primary care. [3] Those presenting with alarm symptoms equating to a CRC risk of ≥3% can be referred via an urgent suspected cancer pathway, following the National Institute for Health and Care Excellence (NICE) NG12 guidelines. [4] Recognising that patients with lower-risk ("low risk, but not no-risk'') [5] symptoms may also have cancer, more recent NICE guidance (DG30) recommends offering a faecal immunochemical test (FIT) to patients "without rectal bleeding who have unexplained symptoms but do not meet criteria for a suspected cancer pathway". [6] Those with results showing blood concentration of ≥10 µg Hb/g faeces are recommended to be referred for further investigations, most often a colonoscopy.

The evidence review that informed DG30 development did not identify any studies using FIT in primary care before referral (i.e. as a triage tool), [7] raising the risk of spectrum bias (variation in performance due to testing in different populations). [8] However, since implementation of this guidance, several studies in primary care (including patients with higher-risk and/or lower-risk symptoms) [9-17] have demonstrated that FIT is an effective tool for CRC in primary care, helping to reassure patients and professionals when results are negative, and optimising referrals for definitive investigations when results are positive. Different FIT thresholds may also be used to indicate patients with different levels of risk. [9, 10, 17] Due to the COVID-19 pandemic, use of FIT has recently been intensified, including to prioritise urgent referrals. [18]

A comprehensive analysis of diagnostic performance of FIT in Southwest England showed that FIT performs exceptionally well to triage patients presenting in primary care with lower-risk CRC symptoms (as specified by DG30 guidance). [9] However, evidence is still limited on how guidance is being used in practice, and for which patients. There is also little evidence about the presenting symptom/s that prompt patients to consult and general practitioners (GPs) to request a FIT. The aim of this study was to investigate use of FIT in English primary care for patients who did not immediately meet criteria for urgent referral. We report on the characteristics of patients for whom a FIT was requested, and agreement between patient-reported and GP-recorded symptoms.

Methods

Design

For peer review only - http://bmjopen.bmj.com/site/about/guidelines.xhtml

We conducted a pragmatic, prospective, descriptive study in the East of England, where a quantitative FIT service for diagnostic triage of lower-risk symptomatic patients was launched in primary care from late 2018.

Setting and population

The study was set in all Eastern Cancer Alliance General Practices in two of six Sustainability and Transformation Plans (STPs): Suffolk & North East Essex and Norfolk & Waveney (estimated adult population of 1,160,215).[19]

We included adult patients aged ≥40 years who were seen by their GPs with symptoms of possible CRC between January and June 2020, but who were not urgently referred for investigation (NICE NG12),[4, 6] and for whom a FIT was requested. We excluded patients receiving a FIT for asymptomatic screening purposes, or patients deemed by GPs as lacking capacity for informed consent.

Two laboratories responsible for all the FITs added study recruitment letters, consent forms and a prepaid envelope to FIT kits distributed to GP practices. These were given to patients when a FIT was requested for patients who did not immediately meet urgent referral criteria.

Data sources

Study data

Data collection started on 24 January 2020 (kits sent from laboratories to GP practices) and ceased on 31 January 2021 (data obtained from primary care records). Patient consent forms were only accepted until 30 June 2020 due to clinical pathway changes resulting from the COVID-19 pandemic (i.e., FIT being increasingly performed in primary care for higher-risk patients who before the pandemic would meet criteria for urgent referral).

Patients were asked about their age, gender and postcode (to calculate deprivation quintiles) on consent forms and invited to complete a questionnaire (Supplementary File 1) by post or online (Qualtrics, Provo, UT). A reminder was sent after two weeks. The questionnaire was developed from the SYMPTOM questionnaire[20-22] and the Family History Questionnaire (FHQ-12).[23, 24] It included items on symptoms (change in bowel habit, change in bowel habit with mainly diarrhoea, change in bowel habit with mainly constipation, abdominal pain, rectal bleeding, indigestion/heartburn, bloating, fatigue, unexplained weight loss, loss of appetite and mucus in faeces), symptom severity (Likert-type items, from mild (1) to unbearable (6)), socio-

For peer review only - http://bmjopen.bmj.com/site/about/guidelines.xhtml

demographic characteristics and lifestyle behaviours, medication use, medical history and family history of CRC.

The laboratories also collected FIT and blood test results on consenting patients. The FIT assays used were OC-Sensor (Norfolk) and HM-JACKarc (Suffolk): a ‘positive’ test was defined as fHb ≥10 µg Hb/g faeces, as per NICE DG30[6]. Results using the OC-Sensor were presented in ng/ml, which were then converted into µg Hb/g by dividing the results by 5. Additionally, laboratories provided GP-recorded symptom data, collected via the FIT request form which had been adapted with the Eastern Cancer Alliance, to match symptoms in the patient questionnaire (except for additional questions about mucus in faeces and symptom severity which were only available for patients).

Finally, primary care records were accessed by practice staff or remotely by the research team (if approved by practices) ≥6 months after FIT was requested to collect clinical outcome data (medical history and cancer diagnoses). These data were only available for a third of patients as practices had to be contacted about each consenting patient – resulting in unfeasible additional workload for many primary care practices during the COVID-19 pandemic. It is unlikely that these data were missing at random and clinical outcome data are only reported descriptively to avoid misleading results – no measures of diagnostic performance were therefore calculated.

Data on patient age, gender and postcode from consent forms were cross-checked when provided by more than one data source (patient responses were prioritised). A database was created in Microsoft Access 2016 (secure server, password protected) for data collection and recording of key data, study timelines and information on data completeness.

Historical data for comparison purposes

Anonymised historical, aggregated data on FIT requests for symptomatic patients, FIT results and GP-recorded symptom/s with FIT request were provided by the North East Essex and Suffolk Pathology Services and the NHS Ipswich and East Suffolk Clinical Commissioning Group. These data covered the period from FIT implementation (test launch in primary care) in November 2018 to May 2020, for the whole of East of England. This was important for comparison purposes, and for assessing relevance of study population/symptomatology.

Data analysis

For peer review only - http://bmjopen.bmj.com/site/about/guidelines.xhtml

Data from consenting patients were anonymised and exported into SPSS v.27 format. Analyses were carried out for patients with data from questionnaires, for whom there were also FIT results and GP-reported symptom data. This ensured a full, complete dataset.

Body mass index (BMI) was calculated using patient self-reported values (kg/m^2). Practice postcodes were used to calculate deprivation when patient postcodes were missing. All other missing data were coded as such, using the same code/label to ensure consistency across different variables and datasets.

Descriptive statistics (frequencies, percentages and measures of central tendency) were used for patient self-reported data, GP-recorded symptoms, symptom severity and test results. Agreement between symptoms reported by patients and recorded by GPs was calculated as the number of agreed upon symptoms (both patient-reported and GP-recorded) divided by the sum of cases with agreements and the cases with disagreements. Kappa statistics were calculated and interpreted as poor or worse than chance (<0.00) slight (0.00-0.20), fair (0.21-0.4), moderate (0.41-0.60), substantial (0.61-0.80) or almost perfect (0.81-1.00) agreement. [25]

Patient and public involvement statement

There was no patient and public involvement in this analysis. However, the study also includes a qualitative component to assess views on FIT experience and acceptability (reported separately) with PPI involvement (specifically, reviewing and analysing a subsection of interview transcripts).

Results

Population and setting

From January to June 2020, 560 patients from in Suffolk & North East Essex and Norfolk & Waveney (average 108 per month) consented to take part in the study.

Historical data (Supplementary file 2) for the first half of 2020 (January to May 2020) show that there were 6287 FIT requests in the whole South East of England (average 1257 per month with variation during the COVID-19 pandemic).

Patient flow

507 consenting patients (91%) were eligible for inclusion (“initial sample”). Linked data from all sources (“full dataset” with patient data (questionnaire), lab records and GP recording of symptoms for each patient) were available for 310 (61%) linked patients (Figure 1).

For peer review only - http://bmjopen.bmj.com/site/about/guidelines.xhtml

[Insert Figure 1 here]

Patient characteristics

In the full dataset (n=310), almost a quarter of FIT results were positive (23%, n=72). Historical data show a similar proportion of positive FITs for the East of England from November 2018 (test launch) to May 2020 (15,620 valid FITs were performed overall, 21% (n=3237) positive) (Supplementary File 2).

Patient median age was 70 (IQR 61-77) and over half were female (53%) (Table 1). About a quarter of patients (26%) lived in areas of higher deprivation. Compared to the initial sample (n=507), the full dataset was representative in terms of gender, but the most deprived and older patients were underrepresented (Supplementary Table 1). Most patients identified as White British (94%), over a third had a Higher Education degree (37%) and two-thirds (66%) reported having had a close relative with CRC prior to the age of 55.

Table 1. Sociodemographic characteristics of sample

	**Full dataset (n=310)**
**Available for all in the initial sample**	
Age	
Median age (IQR)	70 (61-77)
Gender	n (%)
Female	160 (53)
Male	142 (47)
Deprivation (IMD Quintiles)	
1 (least deprived)	70 (23)
2	73 (24)
3	88 (28)
4	49 (16)
5 (most deprived)	30 (10)
**Questionnaire data**	
Ethnicity	n (%)
White British	289 (94)
White non-British[a]	13 (4)
Other[b]	7 (2)
Education	
Higher education	110 (37)
A-Level or equivalent	33 (11)
GCSE/O-level or equivalent	79 (26)
Other[c]	18 (6)
None	60 (20)
Family history	
Close relatives with CRC before the age of 55	17 (66)
More than one relative with CRC	40 (13)

Percentages may not add up to 100% due to rounding. Missing data: gender (full dataset n=8, 2.6%; initial dataset n=12, 2.4%); ethnicity (n=1, 0.3%), education (n=11, 3.2%). Percentages in table calculated considering complete cases. [a]White Irish (n=7), any other White background (n=6). [b]Not further described to protect patient anonymity as n<5 for each). [c]Any other qualification, e.g. "Diploma", "High School", "Police Promotion Exams, "Teacher", etc.

For peer review only - http://bmjopen.bmj.com/site/about/guidelines.xhtml

Table 2 shows that half the sample were non-smokers, 44% were frequent drinkers, over 50% were either overweight or obese, and almost three-quarters had a bowel screening test in the past five years. The most common non-gastrointestinal comorbidities were arthritis and anxiety or depression, and most blood test results were normal.

Table 2. Patient lifestyle, medication use and co-morbidities

	Full dataset (n=310)
	n (%)
**Questionnaire data**	
Smoking status	
Current smoker	23 (7)
Ex-smoker	125 (40)
Non-smoker (never smoked)	162 (52)
Alcohol in last 12 months	
Frequent drinker	138 (45)
Infrequent drinker	48 (16)
Teetotal/rarely drinks	124 (40)
BMI	
Underweight (below 18.5)	12 (4)
Normal (18.5-24.9)	135 (44)
Overweight (25-29.9)	101 (33)
Obese (30 and above)	56 (18)
Imaging and screening	
Bowel screening test (past 5 years)	226 (73)
Medication use [26]	
NSAIDs	59 (19)
Aspirin	47 (15)
Anticoagulants/blood thinners	51 (17)
Antidepressants	38 (12)
None of the above	145 (47)
Co-morbidities (non-GI only)	
Diabetes	43 (14)
Arthritis	95 (31)
Anxiety or depression	77 (25)
**Laboratory data[b]**	
Hb[c](n=282)	
Low	51 (18)
Within normal range	231 (82)
Platelet count[d] (n=282)	
Low	11 (4)
Within normal range	254 (90)
High (>400)	17 (6)
ESR[e] (n=53)	
Low	3 (6)
Within normal range	32 (60)
High	18 (34)
CRP[f] (n=190)	
Within normal range	142 (75)
High	48 (25)

Percentages may not add up to 100% due to rounding. BMI: Body mass index. NSAIDS: Nonsteroidal anti-inflammatory drugs. Hb: Haemoglobin. ESR: Erythrocyte sedimentation rate. CRP: C-Reactive protein. Missing data: BMI (n=6). Percentages in table calculated considering complete cases. [a]Frequent drinker: almost every day or about twice a week. Infrequent drinker: about once a week or about once a fortnight. Teetotal/rarely drinks: only a few times a year or never drinks alcohol.[b]4 cases with available test results were excluded as they were not carried out ≤6 months of a FIT (n=3) or dates were not available (n=1) – these tests are more likely to have been carried out for different reasons. Median interval between FIT and other tests was 2 days (IQR 6.5-). [c]Hb ranges: low <130 males and <115 females, within normal range 130-180 males and 115-165 females. [d]Platelet ranges: low <140, within normal range 140-400, high >400. [e]ESR ranges: low <1 males and <3 females, within normal range 1-10 males and 3-15 females, high >10 males and >5 females. [f]CRP within normal range 0-5, high >5.

For peer review only - http://bmjopen.bmj.com/site/about/guidelines.xhtml

Symptoms reported by patients and recorded by their GPs

Commonly reported symptoms

The commonest patient-reported symptoms were change in bowel habit (69%) and fatigue (57%) while the least common was rectal bleeding (23%). In comparison, the commonest GP-recorded symptoms were abdominal pain (25%) and change in bowel habit (24%) while the least common was loss of appetite (3%) (Table 3). Two hundred different patient-reported symptom patterns were identified; most of them (n=140) were reported only once. The maximum number of times a symptom pattern was reported was nine times (fatigue only), or 11 times if having no symptoms is considered a pattern (Supplementary Figure 1).

Historical data for the East of England also show that abdominal pain (20%) and change in bowel habit (35%) were the commonest GP-recorded symptoms while loss of appetite was also the least common (3%) (Supplementary File 2).

Patient- and GP- agreement

Agreement between patient-reported and GP-recorded symptoms ranged from 44% for fatigue to 80% for unexplained weight loss. Kappa agreement was universally low across symptoms (lowest for bloating, fatigue and loss of appetite). GPs recorded symptoms less often, and recorded fewer symptoms per patient (median 1 symptom, IQR 1-1), compared to patients (median 5, IQR 3-6 – excluding mucus in faeces) (Table 3).

Similarly, historical data for the whole of East of England show that the proportion of GP-recorded symptoms was lower when compared to patient-reported symptoms, for all symptoms (Supplementary File 2).

Table 3. Patient-reported and GP- recorded symptoms and agreement

	Full dataset (n=310)					
	Patient-reported[a]	GP-recorded[b]	Reported by patient but not recorded by GP	Recorded by GP but not reported by patient	Agreement	Kappa (95% CIs)[c]
	n (%)	n (%)	n (%)	n (%)	%	
Change in bowel habit (CIBH)	213 (69)	75 (24)	146 (47)	8 (3)[d]	50	0.167 (0.100 - 0.234)
CIBH: diarrhoea	139 (45)	72 (23)	77 (25)	10 (3)[e]	72	0.406 (0.312 - 0.500)
CIBH: constipation	93 (30)	32 (10)	69 (22)	8 (3)[f]	75	0.272 (0.164 - 0.380)
Abdominal pain	170 (55)	77 (25)	114 (37)	21 (7)	57	0.169 (0.080 - 0.257)
Rectal bleeding	72 (23)	21 (7)	60 (19)	9 (3)	78	0.171 (0.057 - 0.285)

For peer review only - http://bmjopen.bmj.com/site/about/guidelines.xhtml

Indigestion / heartburn	128 (41)	11 (4)	119 (38)	2 (1)	61	0.069 (0.014 - 0.124)
Bloating	170 (55)	16 (5)	158 (50)	4 (1)	48	0.038 (0.005 - 0.081)
Fatigue	178 (57)	35 (11)	158 (50)	15 (5)	44	-0.001 (-0.064 - 0.062)
Unexplained weight loss	76 (25)	23 (7)	57 (18)	4 (1)	80	0.305 (0.187 - 0.423)
Loss of appetite	89 (29)	8 (3)	85 (27)	4 (1)	71	0.037 (-0.0305 - 0.100)
Mucus in faeces	85 (28)	N/A	N/A	N/A	N/A	N/A

Percentages may not add up to 100% due to rounding. [a]Median interval between FIT request being processed by the lab and the patient returning the questionnaire where symptoms were reported was 42 days (IQR 30-56). [b]GPs recorded symptoms when requesting a FIT. [c]Kappa interpretation: poor or worse than chance (<0.00) slight (0.00-0.20), fair (0.21-0.4), moderate (0.41-0.60), substantial (0.61-0.80) or almost perfect (0.81-1.00) agreement. [d]In all these cases patient had not reported change in bowel habit, diarrhoea nor constipation. [e]In five of these cases patient had reported change in bowel habit. [f]In six of these cases patient had reported change in bowel habit. N/A: not applicable

Patient-reported symptom severity

Patients reported that most symptoms (except for change in bowel habit and change in bowel habit-diarrhoea) were usually either mild or moderate. “Severe” and “very severe” symptoms were reported for all types of change in bowel habit more frequently than for other symptoms (Figure 2).

The proportion of GPs recording symptoms often increased based on patient-reported symptom severity. This was particularly evident for rectal bleeding, abdominal pain and unexplained weight loss, although there was variation due to small numbers (particularly very few “very severe” or “unbearable” cases) (Figure 2).

[Insert Figure 2 here]

Clinical outcomes

Clinical outcomes were only available for 105 patients in the full dataset (34%); 26 had a positive FIT result (25%). Three patients received a CRC diagnosis (3% of 105 patients and 12% of FIT positives), comprising two rectal cancers and one carcinoma of the caecum. All CRC cases had positive FIT results with values >400 µg Hb/g faeces. One patient was diagnosed with endometrial cancer (negative FIT result). Twenty-two patients received an alternative gastrointestinal diagnosis, most commonly diverticular disease, diverticulosis or diverticulitis (n=12).

Discussion

This study set in routine English primary care showed that FIT usage for symptomatic patients was greater in females and in older patients - perhaps unsurprisingly as CRC incidence

For peer review only - http://bmjopen.bmj.com/site/about/guidelines.xhtml

increases with age, but the gender balance is different than expected as colorectal cancer is more common in males. [1] Patients reported on more symptoms than GPs recorded when ordering FITs. While GP reporting of symptoms increased based on symptom severity, agreement between patient- and GP-reporting was low suggesting that risk assessment algorithms such as RATs [27] could under-perform. Pre-pandemic we aimed to understand real-life use of FIT for patients presenting in primary care with symptoms that could indicate CRC, particularly those with lower risk symptoms. As the COVID-19 pandemic progressed it became clear that FIT was being used more extensively for patients with higher-risk symptoms, therefore we stopped the study prematurely.

This study provides much needed data on agreement between patient-reported and GP-recorded symptoms prompting a FIT request. To our knowledge, this is the first time that these findings are reported for English populations. While this study was neither designed nor powered to look at cancer outcomes, poor access to primary care records compounded this aspect, and was a key limitation. The analytic approach, creating a full dataset that contained key variables for all patients (symptom data and FIT results) reduced our sample size, with an over-representation of the least deprived and older patients. The study also over-represented the White British population (estimated to be 85.2% in the East of England according to the latest Census). [28] Nonetheless, comparisons with historical data from the whole of East of England showed similar patterns regarding the proportion of positive FITs, and on GP-recorded symptoms. These similarities further validate the relevance of this study. Finally, patients did not provide information on symptoms at the same time as GPs (increasing the risk of recall bias), and different symptom collection methods were used.

Under-recording of symptoms by healthcare professionals is common and has also been previously linked to symptom severity and their perceived importance. [29-34] We identified only one other study describing both patient- and GP-reported symptoms when a FIT is requested in primary care. [34] Set in Swedish primary care, Högberg *et al* also described low symptom agreement, with GPs reporting symptoms less often[34]. In our study, GPs were compelled to tick one or more symptoms from a list to request a FIT; GPs may have chosen to record only the symptoms that triggered clinical action, and/or prioritised what to record. Their clinical interpretation of a symptom (such as its importance or duration) may also differ from the patient perspective. This may explain why GPs reported symptoms which sometimes were not described by patients (often alarm symptoms such as abdominal pain, rectal bleeding and change in bowel habit), and why agreement was highest for unexplained weight loss and rectal bleeding, both alarm symptoms. Some patients may have under-reported their symptoms to their GP, or only mentioned the most severe symptom. Nonetheless, low symptom agreement

For peer review only - http://bmjopen.bmj.com/site/about/guidelines.xhtml

between GPs and patients suggests possible recording bias and sub-optimal record-keeping. These can have implications for patient management, such as the risk of underestimating the importance of certain symptoms for patients' cancer risk assessment and quality of life, although this possibility needs to be further investigated. It is vital that GPs are aware of this discrepancy in symptom reporting/recording; our findings could help to inform education materials for GPs and other health care professionals. Improving accuracy of symptom recording could help GPs to better understand the symptom continuum (i.e. symptoms that persist or worsen over time) and improve efficacy of risk assessment tools embedded in electronic medical records. This not only has crucial implications for detecting cancers in primary care[35], but can also more accurately guide detection of conditions other than cancer.

The Swedish study found the most common patient-reported symptoms were abdominal pain and diarrhoea, in contrast to change in bowel habit and fatigue in our study[34]. Both studies reported the same two most common GP-reported symptoms, abdominal pain and change in bowel habit. These symptoms, alongside age criteria, could potentially trigger an urgent referral in the UK. [4] It may be that GPs assessed these patients as being lower-risk, based on other clinical characteristics. [11, 15] Our results suggest that FIT is being used to aid assessment of "grey area" patients, in line with its initially intended use. However, the high proportion of reported alarm symptoms, alongside older populations, also indicate that FIT was being used beyond its intended use, including for patients previously considered to be higher-risk. This was partly due to new guidance brought in response to the COVID-19 pandemic but cannot wholly be explained by this public health emergency.

Importantly, this wider adoption of FIT for patients with both lower-risk and higher-risk symptoms does not mean that the more recent use is not appropriate. Recent studies do show that FIT has the potential to be used more widely. [9, 10, 17]. Furthermore, recent evidence of the expanded use of FIT in England during the COVID-19 pandemic show a range of positive outcomes, including reduction in mortality[18], significant reduction in the use of endoscopy without compromising CRC detection rates[36], and successful prioritisation of patients with a higher risk of cancer when endoscopy services were severely restricted[37]. GPs also had positive views on the use of FIT during the COVID-19 pandemic[38]. Finally, there is evidence that FIT is seen as an acceptable test by symptomatic patients[39]. Perhaps FIT use has become more embedded and widespread in primary care, and clinicians have become more confident with its use(s). With access to hospital services including endoscopy under pressure during the pandemic, FIT has become a standard filter triage test in vague symptom pathways and is now an integral part of most 2WW colorectal pathways. [40, 41] Furthermore, recent guidance from the Association of Coloproctology of Great Britain & Ireland and the British

For peer review only - http://bmjopen.bmj.com/site/about/guidelines.xhtml

Society of Gastroenterology recommends the use of FIT as a diagnostic triage tool in primary care, including for patients with red flag symptoms such as rectal bleeding. [42, 43]. NICE also plans to publish additional guidance on FIT by November 2023[44]. There is also ongoing work being conducted to support the introduction of using FIT results to risk stratify patients into a 3-tier referral system for full colonoscopy, capsule endoscopy and no further investigation[45]. Future research will be required to assess this expanding role of FIT outside the current NICE referral guidelines. With an increasing reliance on FIT as a triage tool for symptomatic patients in primary care, particular attention should be given to the application of repeat FIT (with optimum intervals and thresholds) [46] and the impact of using different cut-off points[34]: safety-netting will become even more vital to avoid missing cancer cases with a negative FIT result[46-48].

Figure legends

Figure 1. Study flow diagram

[a]16 patients returned more than one questionnaire. The first response was kept to reduce recall bias
GP: general practitioner. FIT: faecal immunochemical test.

Figure 2. Symptom severity reported by patients

Supplementary data

Strobe checklist

Supplementary File 1. Patient questionnaire

Supplementary File 2. FIT requests and GP-recorded symptoms in the South East of England between November 2018 and May 2020

Supplementary Figure 1. Patient-reported symptom patterns

Supplementary Table 1. Patient characteristics according to availability of linked data

Additional information

Data sharing statement

Anonymised data are available from the authors upon reasonable request.

Funding

This research arises from the CanTest Collaborative, which is funded by Cancer Research UK C8640/A23385, of which Fiona M Walter and Willie Hamilton are Directors, Niek de Wit is an Associate Director, Natalia Calanzani and Merel M Pannebakker are postdoctoral researchers. The funders of the study had no role in study design, data collection, data analysis, data interpretation, or writing of the report.

Ethical approval

For peer review only - http://bmjopen.bmj.com/site/about/guidelines.xhtml

Ethical approval for the FIT-East study was granted by the East of England Cambridge Central Research Ethics Committee – reference 19/EE/0036.

Competing interests

None declared.

Patient and public involvement

There was no PPI involvement in the quantitative component of the study reported here.

Patient consent for publication

Not applicable.

Contributors

MMP and FMW planned the original study; WH and FMW secured funding. Analysis was planned by MMP, NC, MJT, HW, NdW, WH, PH and FMW and carried out by NC, MJT, HW and FMW. NC drafted the manuscript with senior input from WH, NdW and FMW and revised it after critical reviews from all authors. All authors read and approved the final manuscript.

Acknowledgements

We thank all the patients who agreed to take part in the study, and who completed the questionnaire. We thank the general practice staff who provided us with patient outcome data. We thank Allison Chipchase, Eve Calderbank and Claire Corbett for their help obtaining historical data. We thank the North East Essex and Suffolk Pathology Services at the East Suffolk and North Essex NHS Foundation Trust, and the Clinical Biochemistry section of Laboratory Medicine at Norfolk and Norwich University Hospitals NHS Foundation Trust for all their help providing laboratory data. We are grateful to Dr Marije van Melle for her work in the early stages of study design, and to Dr Sarah ER Bailey for her help during protocol development. We are grateful to Dr Lina Massou for expert statistical input. We thank Aina Chang and Sara Shaida for their help with data collection and gathering background information for the study, James Brimicombe for data management advice, support and expertise. We are indebted to Andy Cowan for his invaluable support collecting and managing study data, and for his comments on the study results. We also thank Victoria Hardy, Dr Owain T Jones and Dr Yin Zhou for their comments on the study results.

References

1. GLOBOCAN 2018. Estimated number of deaths worldwide, both sexes, all ages 2020 [Available from: https://gco.iarc.fr/].

For peer review only - http://bmjopen.bmj.com/site/about/guidelines.xhtml

2. National Cancer Registration and Analysis Service (NCRAS). Percentage of Diagnoses by route - 2006 to 2016. In: (a) RtD-w, editor. Microsoft Excel2019.
3. Koo MM, von Wagner C, Abel GA, McPhail S, Hamilton W, Rubin GP, et al. The nature and frequency of abdominal symptoms in cancer patients and their associations with time to help-seeking: evidence from a national audit of cancer diagnosis. Journal of Public Health. 2018;40(3):e388-e95.
4. NICE. Suspected cancer: recognition and referral NICE guideline [NG12] 2015 [Available from: https://www.nice.org.uk/guidance/ng12].
5. Hamilton W. The CAPER studies: five case-control studies aimed at identifying and quantifying the risk of cancer in symptomatic primary care patients. Br J Cancer. 2009;101 Suppl 2(Suppl 2):S80-S6.
6. NICE. Quantitative faecal immunochemical tests to guide referral for colorectal cancer in primary care (DG30) 2017 [Available from: https://www.nice.org.uk/guidance/dg30].
7. Westwood M, Corro Ramos I, Lang S, Luyendijk M, Zaim R, Stirk L, et al. Faecal immunochemical tests to triage patients with lower abdominal symptoms for suspected colorectal cancer referrals in primary care: a systematic review and cost-effectiveness analysis. Health Technol Assess. 2017;21(33):1-234.
8. Walter FM, Thompson MJ, Wellwood I, Abel GA, Hamilton W, Johnson M, et al. Evaluating diagnostic strategies for early detection of cancer: the CanTest framework. BMC Cancer. 2019;19(1):586.
9. Bailey SER, Abel GA, Atkins A, Byford R, Davies SJ, Mays J, et al. Diagnostic performance of a faecal immunochemical test for patients with low-risk symptoms of colorectal cancer in primary care: an evaluation in the South West of England. Br J Cancer. 2021.
10. Digby J, Strachan JA, McCann R, Steele RJ, Fraser CG, Mowat C. Measurement of faecal haemoglobin with a faecal immunochemical test can assist in defining which patients attending primary care with rectal bleeding require urgent referral. Annals of Clinical Biochemistry. 2020;57(4):325-7.
11. Nicholson BD, James T, Paddon M, Justice S, Oke JL, East JE, et al. Faecal immunochemical testing for adults with symptoms of colorectal cancer attending English primary care: a retrospective cohort study of 14 487 consecutive test requests. Alimentary Pharmacology & Therapeutics. 2020;17:17.
12. Chapman C, Thomas C, Morling J, Tangri A, Oliver S, Simpson JA, et al. Early clinical outcomes of a rapid colorectal cancer diagnosis pathway using faecal immunochemical testing in Nottingham. Colorectal Disease. 2020;22(6):679-88.
13. Hogberg C, Gunnarsson U, Cronberg O, Thulesius H, Lilja M, Jansson S. Qualitative faecal immunochemical tests (FITs) for diagnosing colorectal cancer in patients with histories of rectal bleeding in primary care: a cohort study. International Journal of Colorectal Disease. 2020;35(11):2035-40.
14. Hogberg C, Gunnarsson U, Jansson S, Thulesius H, Cronberg O, Lilja M. Diagnosing colorectal cancer in primary care: cohort study in Sweden of qualitative faecal immunochemical tests, haemoglobin levels, and platelet counts. British Journal of General Practice. 2020;70(701):e843-e51.
15. Juul JS, Hornung N, Andersen B, Laurberg S, Olesen F, Vedsted P. The value of using the faecal immunochemical test in general practice on patients presenting with non-alarm symptoms of colorectal cancer. Br J Cancer. 2018;119(4):471-9.
16. Mowat C, Digby J, Strachan JA, McCann R, Hall C, Heather D, et al. Impact of introducing a faecal immunochemical test (FIT) for haemoglobin into primary care on the outcome of patients with new bowel symptoms: a prospective cohort study. BMJ Open Gastroenterology. 2019;6(1):e000293.
17. Pin-Vieito N, Tejido-Sandoval C, de Vicente-Bielza N, Sánchez-Gómez C, Cubiella J. Faecal immunochemical tests safely enhance rational use of resources during the assessment of suspected symptomatic colorectal cancer in primary care: systematic review and meta-analysis. Gut. 2021:gutjnl-2021-324856.

For peer review only - http://bmjopen.bmj.com/site/about/guidelines.xhtml

18. Loveday C, Sud A, Jones ME, Broggio J, Scott S, Gronthound F, et al. Prioritisation by FIT to mitigate the impact of delays in the 2-week wait colorectal cancer referral pathway during the COVID-19 pandemic: a UK modelling study. Gut. 2020;27:27.
19. Office for National Statistics. Table SAPE22DT6a: Mid-2019 Population Estimates for 2020 Clinical Commissioning Groups in England by Single Year of Age and Sex, Persons - National Statistics 2020 [Available from: https://www.ons.gov.uk/peoplepopulationandcommunity/populationandmigration/populationestimates/datasets/clinicalcommissioninggroupmidyearpopulationestimates.
20. Walter FM, Emery JD, Mendonca S, Hall N, Morris HC, Mills K, et al. Symptoms and patient factors associated with longer time to diagnosis for colorectal cancer: results from a prospective cohort study. Br J Cancer. 2016;115(5):533-41.
21. Walter FM, Mills K, Mendonça SC, Abel GA, Basu B, Carroll N, et al. Symptoms and patient factors associated with diagnostic intervals for pancreatic cancer (SYMPTOM pancreatic study): a prospective cohort study. Lancet Gastroenterol Hepatol. 2016;1(4):298-306.
22. Walter FM, Rubin G, Bankhead C, Morris HC, Hall N, Mills K, et al. Symptoms and other factors associated with time to diagnosis and stage of lung cancer: a prospective cohort study. Br J Cancer. 2015;112 Suppl 1(Suppl 1):S6-13.
23. Emery JD, Reid G, Prevost AT, Ravine D, Walter FM. Development and validation of a family history screening questionnaire in Australian primary care. Ann Fam Med. 2014;12(3):241-9.
24. Walter FM, Prevost AT, Birt L, Grehan N, Restarick K, Morris HC, et al. Development and evaluation of a brief self-completed family history screening tool for common chronic disease prevention in primary care. Br J Gen Pract. 2013;63(611):e393-400.
25. Landis JR, Koch GG. The measurement of observer agreement for categorical data. Biometrics. 1977;33(1):159-74.
26. Sultan M, Abebe Y, Tsadik AW, Ababa A, Yesus AG, Mould-Millman N-K. Trends and barriers of emergency medical service use in Addis Ababa; Ethiopia. BMC emergency medicine. 2019;19(1):28.
27. ERICA TRIAL. https://www.theericatrial.co.uk/2022 [
28. Office for National Statistics. England and Wales 2011 Census. Population by ethnicity and region 2018 [Available from: https://www.ethnicity-facts-figures.service.gov.uk/uk-population-by-ethnicity/national-and-regional-populations/regional-ethnic-diversity/latest/downloads/population-by-ethnicity-and-region.csv.
29. Chidambaram S, DeShields T, Potter P, Olsen S, Chen L. Patient and provider concordance on symptoms during the oncology outpatient clinic visit. J Community Support Oncol. 2014;12(10):370-7.
30. Valikodath NG, Newman-Casey PA, Lee PP, Musch DC, Niziol LM, Woodward MA. Agreement of Ocular Symptom Reporting Between Patient-Reported Outcomes and Medical Records. JAMA Ophthalmol. 2017;135(3):225-31.
31. Horvath Walsh LE, Rider A, Piercy J, Pike J, Wilson S, Pandya BJ, et al. Real-World Impact of Physician and Patient Discordance on Health-Related Quality of Life in US Patients with Acute Myeloid Leukemia. Oncol Ther. 2019;7(1):67-81.
32. Strömgren AS, Groenvold M, Pedersen L, Olsen AK, Spile M, Sjøgren P. Does the medical record cover the symptoms experienced by cancer patients receiving palliative care? A comparison of the record and patient self-rating. J Pain Symptom Manage. 2001;21(3):189-96.
33. Pakhomov SV, Jacobsen SJ, Chute CG, Roger VL. Agreement between patient-reported symptoms and their documentation in the medical record. Am J Manag Care. 2008;14(8):530-9.
34. Högberg C, Karling P, Rutegård J, Lilja M. Patient-reported and doctor-reported symptoms when faecal immunochemical tests are requested in primary care in the diagnosis of colorectal cancer and inflammatory bowel disease: a prospective study. BMC Family Practice. 2020;21(1):129.

For peer review only - http://bmjopen.bmj.com/site/about/guidelines.xhtml

35. Vedsted P, Olesen F. A differentiated approach to referrals from general practice to support early cancer diagnosis - the Danish three-legged strategy. Br J Cancer. 2015;112 Suppl 1(Suppl 1):S65-9.
36. Kamel F, Zulfiqar S, Penfold W, Weatherell S, Madani R, Nisar P, et al. The use of the faecal immunochemical test during the COVID-19 pandemic to triage urgent colorectal cancer referrals. Colorectal Disease. 2022;24(6):727-36.
37. Archer T, Aziz I, Kurien M, Knott V, Ball A. Prioritisation of lower gastrointestinal endoscopy during the COVID-19 pandemic: outcomes of a novel triage pathway. Frontline Gastroenterology. 2022;13(3):225.
38. Archer S, Calanzani N, Honey S, Johnson M, Neal R, Scott SE, et al. Impact of the COVID-19 pandemic on cancer assessment in primary care: a qualitative study of GP views. BJGP Open. 2021;5(4):BJGPO.2021.0056.
39. Georgiou Delisle T, Souza N, Davies B, Benton S, Chen M, Ward H, et al. Faecal immunochemical test for suspected colorectal cancer symptoms: patient survey of usability and acceptability. BJGP Open. 2022;6(1):BJGPO.2021.0102.
40. NHS England. Clinical guide for triaging patients with lower gastrointestinal symptoms 2020 [Available from: https://www.nice.org.uk/Media/Default/About/COVID-19/Specialty-guides/triaging-patients-with-lower-gi-symptoms.pdf (accessed 20 Jul 2021).
41. Turvill JL, Turnock D, Cottingham D, Haritakis M, Jeffery L, Girdwood A, et al. The Fast Track FIT study: diagnostic accuracy of faecal immunochemical test for haemoglobin in patients with suspected colorectal cancer. British Journal of General Practice. 2021:BJGP.2020.1098.
42. Guideline Development Group (GDG). Faecal Immunochemical Testing (FIT) in patients with signs or symptoms of suspected colorectal cancer (CRC): A joint guideline from the Association of Coloproctology of Great Britain & Ireland (ACPGBI) and the British Society of Gastroenterology (BSG) https://www.bsg.org.uk/clinical-resource/faecal-immunochemical-testing-fit-in-patients-with-signs-or-symptoms-of-suspected-colorectal-cancer-crc-a-joint-guideline-from-the-acpgbi-and-the-bsg/: Association of Coloproctology of Great Britain & Ireland (ACPGBI) and the British Society of Gastroenterology (BSG); 2022 [
43. Monahan KJ, Davies MM, Abulafi M, Banerjea A, Nicholson BD, Arasaradnam R, et al. Faecal immunochemical testing (FIT) in patients with signs or symptoms of suspected colorectal cancer (CRC): a joint guideline from the Association of Coloproctology of Great Britain and Ireland (ACPGBI) and the British Society of Gastroenterology (BSG). Gut. 2022:gutjnl-2022-327985.
44. National Institute for Health and Care Excellence. Quantitative Faecal Immunochemical Tests to guide colorectal cancer pathway referral for people with a change in bowel habit or abdominal pain - GID-DG10036 2022 [Available from: https://www.nice.org.uk/guidance/indevelopment/gid-dg10036.
45. Norfolk and Norwich University Hospitals. Guideline for the Triage and Diagnostic Investigation of Patients Referred with Suspected Colorectal Cancer www.nnuh.nhs.uk2020 [
46. Benton SC, Fraser CG. Faecal immunochemical tests in the COVID-19 pandemic; safety-netting of patients with symptoms and low faecal haemoglobin concentration - can a repeat test be used? Ann Clin Biochem. 2021;58(3):163-5.
47. Craig M, Turner J, Torkington J, Crosby T. Faecal immunochemical test: challenges and opportunities for cancer diagnosis in primary care. British Journal of General Practice. 2022;72(721):366.
48. Laszlo HE, Seward E, Ayling RM, Lake J, Malhi A, Stephens C, et al. Faecal immunochemical test for patients with 'high-risk' bowel symptoms: a large prospective cohort study and updated literature review. Br J Cancer. 2022;126(5):736-43.

For peer review only

For peer review only - http://bmjopen.bmj.com/site/about/guidelines.xhtml

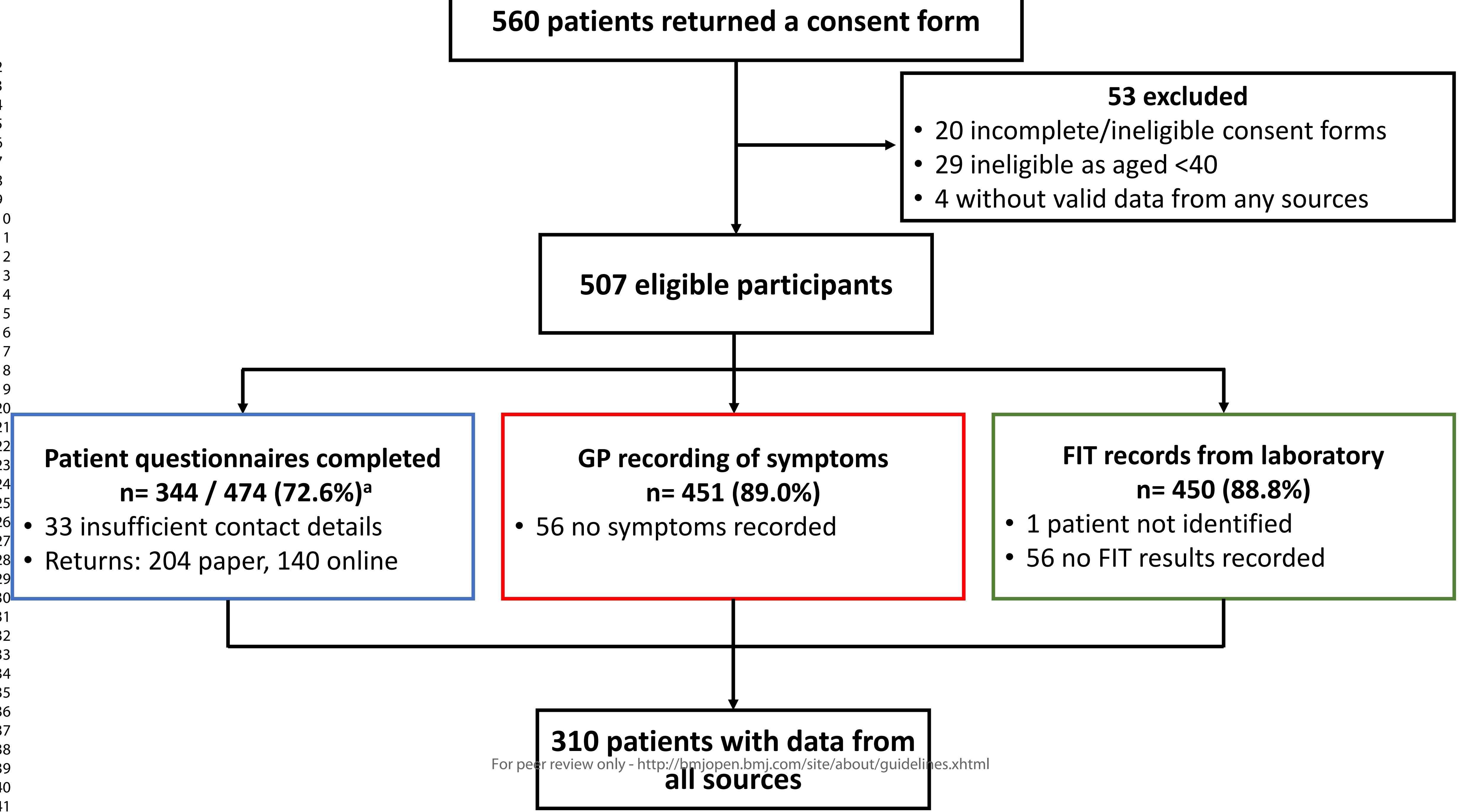
560 patients returned a consent form
53 excluded
• 20 incomplete/ineligible consent forms
• 29 ineligible as aged <40
• 4 without valid data from any sources
507 eligible participants
Patient questionnaires completed
n= 344 / 474 (72.6%)[a]
• 33 insufficient contact details
• Returns: 204 paper, 140 online
GP recording of symptoms
n= 451 (89.0%)
• 56 no symptoms recorded
FIT records from laboratory
n= 450 (88.8%)
• 1 patient not identified
• 56 no FIT results recorded
310 patients with data from all sources

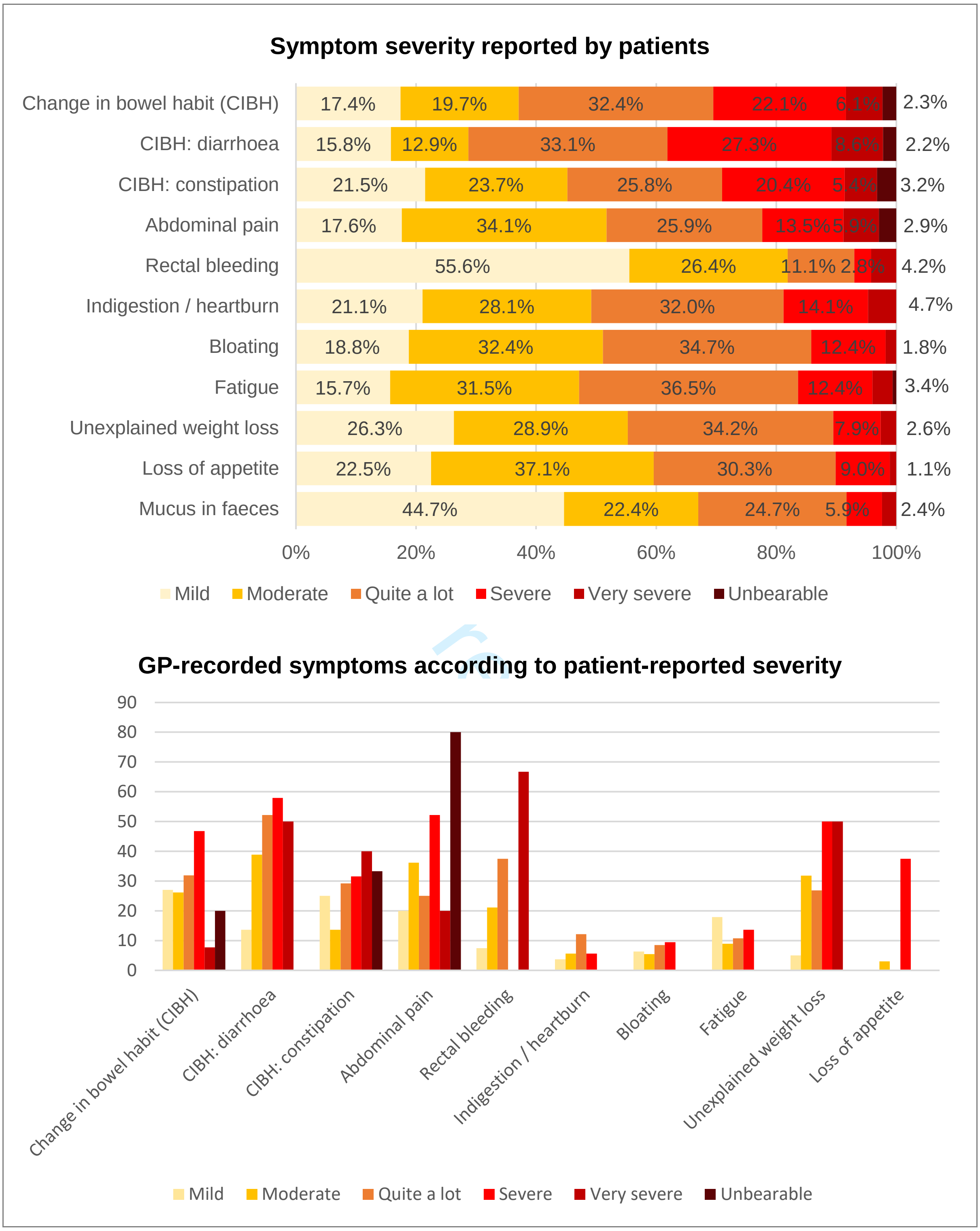
Symptom severity reported by patients
Change in bowel habit (CIBH)
CIBH: diarrhoea
CIBH: constipation
Abdominal pain
Rectal bleeding
Indigestion / heartburn
Bloating
Fatigue
Unexplained weight loss
Loss of appetite
Mucus in faeces
17.4% 19.7% 32.4% 22.1% 6.1% 2.3%
15.8% 12.9% 33.1% 27.3% 8.6% 2.2%
21.5% 23.7% 25.8% 20.4% 5.4% 3.2%
17.6% 34.1% 25.9% 13.5% 5.9% 2.9%
55.6% 26.4% 11.1% 2.8% 4.2%
21.1% 28.1% 32.0% 14.1% 4.7%
18.8% 32.4% 34.7% 12.4% 1.8%
15.7% 31.5% 36.5% 12.4% 3.4%
26.3% 28.9% 34.2% 7.9% 2.6%
22.5% 37.1% 30.3% 9.0% 1.1%
44.7% 22.4% 24.7% 5.9% 2.4%
0% 20% 40% 60% 80% 100%
Mild Moderate Quite a lot Severe Very severe Unbearable
GP-recorded symptoms according to patient-reported severity
0 10 20 30 40 50 60 70 80 90
Change in bowel habit (CIBH)
CIBH: diarrhoea
CIBH: constipation
Abdominal pain
Rectal bleeding
Indigestion / heartburn
Bloating
Fatigue
Unexplained weight loss
Loss of appetite
Mild Moderate Quite a lot Severe Very severe Unbearable

Supplementary data

Who are the patients being offered the faecal immunochemical test in routine English general practice? A prospective descriptive study

Supplementary File 1. Patient questionnaire

Section 1: About Your Symptoms
We are interested in symptoms which you think are related to your recent poo test request. Did you have any of the following symptoms or health problems?

1. Change in bowel habit
☐ Yes ☐ No   If YES please indicate the severity:
☐ Mild ☐ Moderate ☐ Quite a lot ☐ Severe ☐ Very severe ☐ Unbearable

2. Change in bowel habit – mainly diarrhoea or loose poo
☐ Yes ☐ No   If YES please indicate the severity:
☐ Mild ☐ Moderate ☐ Quite a lot ☐ Severe ☐ Very severe ☐ Unbearable

3. Change in bowel habit – mainly constipation
☐ Yes ☐ No   If YES please indicate the severity:
☐ Mild ☐ Moderate ☐ Quite a lot ☐ Severe ☐ Very severe ☐ Unbearable

4. Abdominal pain or discomfort that wasn't normal for you
☐ Yes ☐ No   If YES please indicate the severity:
☐ Mild ☐ Moderate ☐ Quite a lot ☐ Severe ☐ Very severe ☐ Unbearable

5. Bleeding from your back passage
☐ Yes ☐ No   If YES please indicate the severity:
☐ Mild ☐ Moderate ☐ Quite a lot ☐ Severe ☐ Very severe ☐ Unbearable

6. Indigestion or heartburn
☐ Yes ☐ No   If YES please indicate the severity:
☐ Mild ☐ Moderate ☐ Quite a lot ☐ Severe ☐ Very severe ☐ Unbearable

7. Bloating
☐ Yes ☐ No   If YES please indicate the severity:
☐ Mild ☐ Moderate ☐ Quite a lot ☐ Severe ☐ Very severe ☐ Unbearable

8. Fatigue or tiredness that is unusual for you
☐ Yes ☐ No   If YES please indicate the severity:
☐ Mild ☐ Moderate ☐ Quite a lot ☐ Severe ☐ Very severe ☐ Unbearable

9. Unexplained weight loss
☐ Yes ☐ No   If YES please indicate the severity:
☐ Mild ☐ Moderate ☐ Quite a lot ☐ Severe ☐ Very severe ☐ Unbearable

10. Loss of appetite
☐ Yes ☐ No   If YES please indicate the severity:
☐ Mild ☐ Moderate ☐ Quite a lot ☐ Severe ☐ Very severe ☐ Unbearable

11. Mucous or slime in poo
☐ Yes ☐ No   If YES please indicate the severity:
☐ Mild ☐ Moderate ☐ Quite a lot ☐ Severe ☐ Very severe ☐ Unbearable

Section 2: About You
12. How would you describe your ethnicity? Please tick one box only
White

For peer review only

☐ British ☐ Irish
☐ Any other white background. Please write...

Mixed
☐ White and Black Caribbean ☐ White and Black African ☐ White and Asian
☐ Any other Mixed background. Please write...

Chinese or other ethnic group
☐ Chinese ☐ Any other ethnic group. Please write..

Asian or Asian British
☐ Indian ☐ Pakistani ☐ Bangladeshi ☐ Any other Asian background. Please write...............................

Black or Black British
☐ Caribbean ☐ African ☐ Any other Black background. Please write..

13. What is your highest level of qualification? Please tick one box only
☐ Degree (or equivalent) ☐ Other higher education ☐ A-Level (or equivalent)
☐ GCSE/O-Level (or equivalent) ☐ None ☐ Other, please specify................................

14. Do you use any of the following medications?
☐ NSAID/Anti-inflammatory painkillers ☐ Aspirin
☐ Anticoagulants/blood thinners (e.g. warfarin, coumarines) ☐ Antidepressants

15. Are you suffering from, or have you suffered from, any of the following? Please tick any that are applicable to you
☐ Inflammatory bowel disease (e.g. Crohn's or colitis) ☐ Diabetes o Irritable bowel syndrome
☐ Arthritis ☐ Peptic ulcer ☐ Anxiety or depression o Bowel (colon or rectal) cancer ☐ Bowel polyps
☐ Other cancer. Please write which type..

16. Have you had a colonoscopy, sigmoidoscopy or bowel scope?
In the past 5 years? ☐ Yes ☐ No
Ever? ☐ Yes ☐ No

17. Have you had a bowel screening test or poo test?
In the past 5 years? ☐ Yes ☐ No
Ever? ☐ Yes ☐ No

18. Smoking - please tick one box only. Are you?
☐ A current smoker? ☐ An ex-smoker? ☐ A non-smoker (never smoked)?

19. Alcohol. How often have you had an alcoholic drink of any kind in the last 12 months? Please tick one
☐ Almost every day ☐ About once a week ☐ About twice a week ☐ About once a fortnight
☐ Only a few times a year ☐ I never drink alcohol

20. What is your height (fill in one of the options)?
In Feet and Inches or CM...

21. What is your current weight (fill in one of the options)?
Stones and pounds...Kg..

22. Have any of your close relatives, including parent(s), children, brother(s) or sister(s), had colon or rectal cancer (also known as large bowel or colorectal cancer) before the age of 55?
☐ Yes ☐ No

23. Do you have more than one relative who has had colon or rectal cancer on either your mother's or father's side of the family? Please think about all of the following relatives: parent(s), children, brother(s), sister(s), grandparent(s), aunt(s), uncle(s), niece(s), nephew(s) and grandchildren.
☐ Yes ☐ No

For peer review only

Supplementary File 2. FIT requests and GP-recorded symptoms in the South East of England between November 2018 and May 2020

Anonymised data on FIT requests, FIT results and GP-recorded symptom/s with FIT request covering the period from FIT implementation in November 2018 to May 2020.

From November 2018 to May 2020, 15,620 valid FITs were performed in this region. The number of FITs ordered increased from November 2018 to May 2020, reaching a peak in January 2020 (1512 valid tests), reducing during the early pandemic (compared with January, March, 26.3% and April, 50.1% decreases respectively), and increasing again in May 2020 (1465 valid tests) (Figure 1). Of the 15,620 valid FITs performed during this period, 20.7% (n=3237) were positive (range 15.1% (November 2018, Norfolk only) to 29.1% (both areas, April 2020)).

Figure 1. All FIT requests and FIT positives in Eastern England post DG30 implementation

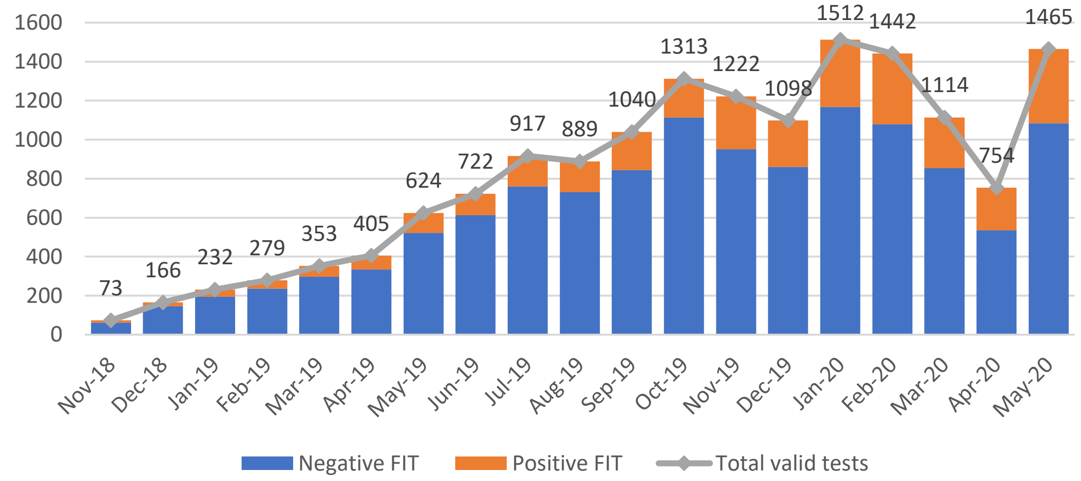

The proportion of GP-recorded symptoms was lower when compared to patient-reported symptoms, and similar to GP-recorded symptoms in the study. This was the case for all reported symptoms (Figure 2).

Figure 2. Historical and FIT-East data for symptom reporting

For peer review only - http://bmjopen.bmj.com/site/about/guidelines.xhtml

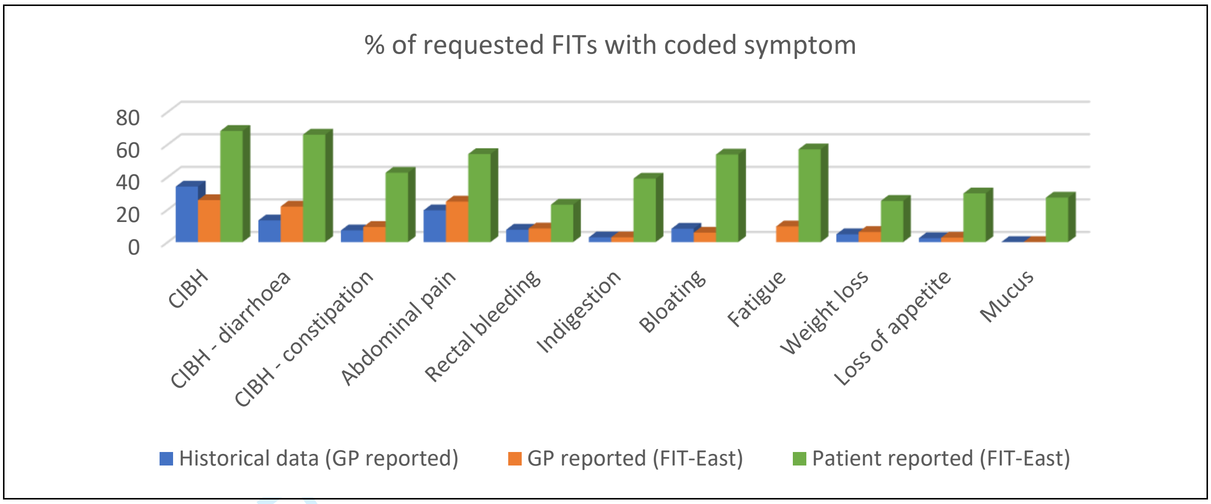

Historical, aggregated data has no information on fatigue, and mucus was only available as an answer option in the patient questionnaire.

For peer review only

Supplementary Table 1. Patient characteristics according to availability of linked data

**Characteristics**	**Linked data available (full dataset) (n=310)**	**Linked data not available (n=197)**	**Tests**
Age			
Median age (IQR)	70 (61-77)	66 (53.5-75.0)	**U=36222.5, *p*<0.001**
Gender	n (%)	n (%)	
Female	160 (53)	100 (52)	
Male	142 (47)	93 (48)	χ(1)=0.064, *p*=0.80
Deprivation (IMD Quintiles)	n (%)	n (%)	
1 (least deprived)	69 (22)	30 (18)	**U=22457, *p*=0.04**
2	74 (24)	26 (16)	
3	88 (28)	55 (34)	
4	49 (16)	34 (21)	
5 (most deprived)	30 (10)	18 (11)	

For peer review only

Supplementary Figure 1. Patient-reported symptom patterns

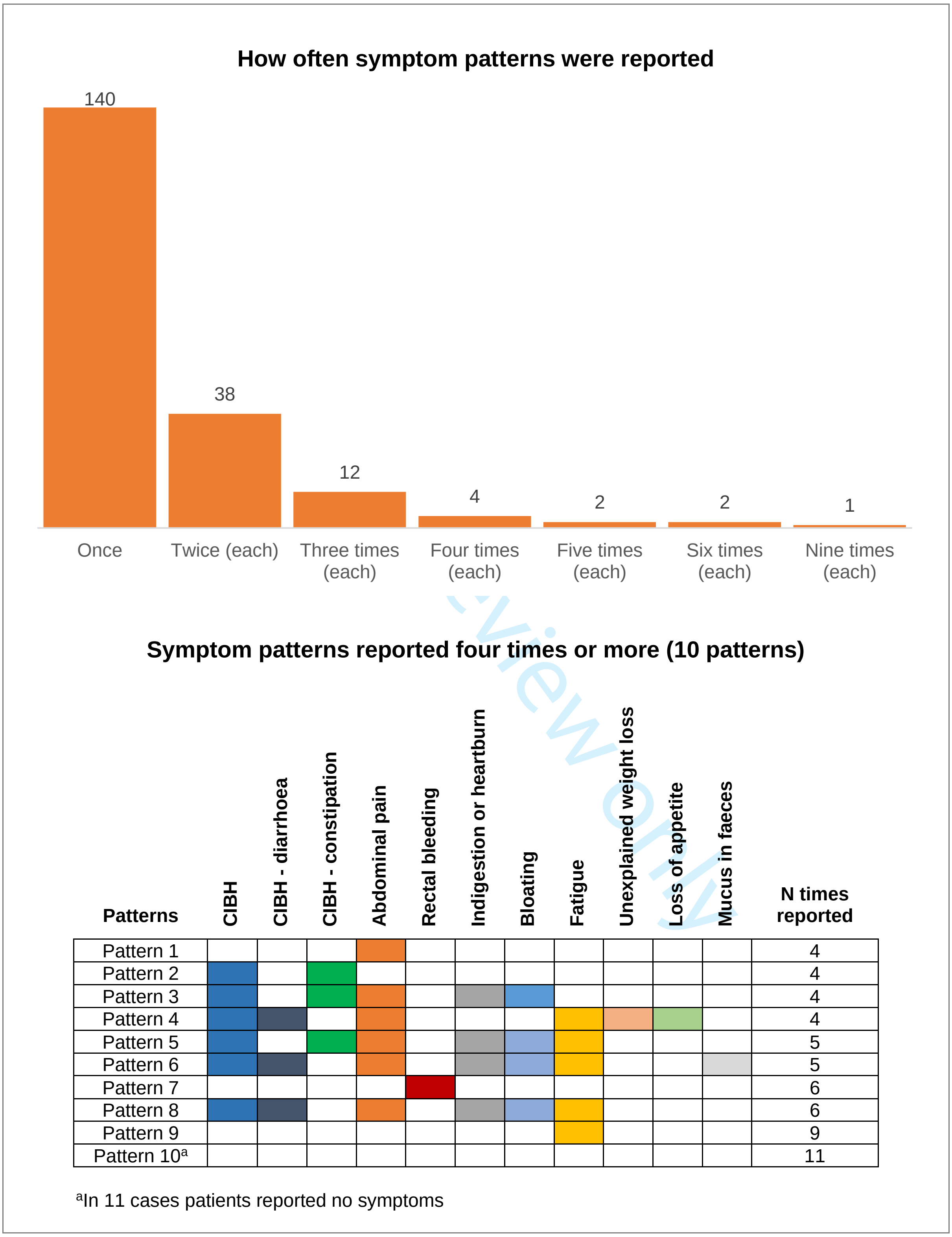

STROBE Statement—checklist of items that should be included in reports of observational studies

	Item No	Recommendation	Page No
**Title and abstract**	1	(*a*) Indicate the study's design with a commonly used term in the title or the abstract	1
		(*b*) Provide in the abstract an informative and balanced summary of what was done and what was found	2
**Introduction**			
Background/rationale	2	Explain the scientific background and rationale for the investigation being reported	4
Objectives	3	State specific objectives, including any prespecified hypotheses	4
**Methods**			
Study design	4	Present key elements of study design early in the paper	4
Setting	5	Describe the setting, locations, and relevant dates, including periods of recruitment, exposure, follow-up, and data collection	5
Participants	6	(*a*) *Cohort study*—Give the eligibility criteria, and the sources and methods of selection of participants. Describe methods of follow-up *Case-control study*—Give the eligibility criteria, and the sources and methods of case ascertainment and control selection. Give the rationale for the choice of cases and controls *Cross-sectional study*—Give the eligibility criteria, and the sources and methods of selection of participants	5-6
		(*b*) *Cohort study*—For matched studies, give matching criteria and number of exposed and unexposed *Case-control study*—For matched studies, give matching criteria and the number of controls per case	N/A
Variables	7	Clearly define all outcomes, exposures, predictors, potential confounders, and effect modifiers. Give diagnostic criteria, if applicable	6-7
Data sources/ measurement	8*	For each variable of interest, give sources of data and details of methods of assessment (measurement). Describe comparability of assessment methods if there is more than one group	5-7, footnote in Table 2
Bias	9	Describe any efforts to address potential sources of bias	Page 6, choice of methods for outcome data
Study size	10	Explain how the study size was arrived at	As above; this was an exploratory descriptive study
Quantitative variables	11	Explain how quantitative variables were handled in the analyses. If applicable, describe which groupings were chosen and why	6-7
Statistical methods	12	(*a*) Describe all statistical methods, including those used to control for confounding	6-7
		(*b*) Describe any methods used to examine subgroups and interactions	6-7
		(*c*) Explain how missing data were addressed	6-7
		(*d*) *Cohort study*—If applicable, explain how loss to follow-up was addressed *Case-control study*—If applicable, explain how matching of cases and controls was addressed	N/A

For peer review only - http://bmjopen.bmj.com/site/about/guidelines.xhtml

		Cross-sectional study—If applicable, describe analytical methods taking account of sampling strategy	
		(*e*) Describe any sensitivity analyses	N/A
**Results**			
Participants	13*	(a) Report numbers of individuals at each stage of study—eg numbers potentially eligible, examined for eligibility, confirmed eligible, included in the study, completing follow-up, and analysed	7, Figure 1
		(b) Give reasons for non-participation at each stage	7, Figure 1
		(c) Consider use of a flow diagram	Figure 1
Descriptive data	14*	(a) Give characteristics of study participants (eg demographic, clinical, social) and information on exposures and potential confounders	7-11, Tables 1-3, Figure 2
		(b) Indicate number of participants with missing data for each variable of interest	Footnote in Tables 1 and 2, "clinical outcomes" subsection (p.11)
		(c) *Cohort study*—Summarise follow-up time (eg, average and total amount)	N/A
Outcome data	15*	*Cohort study*—Report numbers of outcome events or summary measures over time	*N/A*
		Case-control study—Report numbers in each exposure category, or summary measures of exposure	*N/A*
		Cross-sectional study—Report numbers of outcome events or summary measures	7-11, "clinical outcomes" subsection (p.11)
Main results	16	(*a*) Give unadjusted estimates and, if applicable, confounder-adjusted estimates and their precision (eg, 95% confidence interval). Make clear which confounders were adjusted for and why they were included	N/A
		(*b*) Report category boundaries when continuous variables were categorized	Footnote in Table 2
		(*c*) If relevant, consider translating estimates of relative risk into absolute risk for a meaningful time period	N/A
Other analyses	17	Report other analyses done—eg analyses of subgroups and interactions, and sensitivity analyses	N/A
**Discussion**			
Key results	18	Summarise key results with reference to study objectives	11
Limitations	19	Discuss limitations of the study, taking into account sources of potential bias or imprecision. Discuss both direction and magnitude of any potential bias	12
Interpretation	20	Give a cautious overall interpretation of results considering objectives, limitations, multiplicity of analyses, results from similar studies, and other relevant evidence	12-13
Generalisability	21	Discuss the generalisability (external validity) of the study results	12-13
**Other Information**			
Funding	22	Give the source of funding and the role of the funders for the present study and, if applicable, for the original study on which the present article is based	Funding statement, pages 13-14

*Give information separately for cases and controls in case-control studies and, if applicable, for exposed and unexposed groups in cohort and cross-sectional studies.

For peer review only

Note: An Explanation and Elaboration article discusses each checklist item and gives methodological background and published examples of transparent reporting. The STROBE checklist is best used in conjunction with this article (freely available on the Web sites of PLoS Medicine at http://www.plosmedicine.org/, Annals of Internal Medicine at http://www.annals.org/, and Epidemiology at http://www.epidem.com/). Information on the STROBE Initiative is available at www.strobe-statement.org.

For peer review only

For peer review only - http://bmjopen.bmj.com/site/about/guidelines.xhtml
